# V–Ti-Based Solid Solution Alloys for Solid-State Hydrogen Storage

**DOI:** 10.1007/s40820-025-01672-w

**Published:** 2025-03-04

**Authors:** Shaoyang Shen, Yongan Li, Liuzhang Ouyang, Lan Zhang, Min Zhu, Zongwen Liu

**Affiliations:** 1 School of Materials Science and Engineering and Key Laboratory of Advanced Energy Storage Materials of Guangdong Province, South China University of Technology, Guangzhou, 510641 People’s Republic of China; 2 Energy Research Institute at NTU (ERI@N), Nanyang Technological University, 1 CleanTech Loop, Singapore, 637141 Singapore; 3 China-Singapore International Joint Research Institute (CSIJRI), Guangzhou, 510530 People’s Republic of China; 4 Institute of Fundamental and Frontier Sciences, University of Electronic Science and Technology of China, Chengdu, 610054 People’s Republic of China; 5 School of Chemical and Biomolecuar Engineering, The University of Sydney, Sydney, NSW 2006 Australia

**Keywords:** Hydrogen storage, V–Ti-based solid solution alloys, Metal hydride tank, Hydrogen storage properties, Cyclic stability

## Abstract

This review details the advancement in the development of V–Ti-based hydrogen storage materials for using in metal hydride (MH) tanks to supply hydrogen to fuel cells at relatively ambient temperatures and pressures. V–Ti-based solid solution alloys are excellent hydrogen storage materials among many metal hydrides due to their high reversible hydrogen storage capacity which is over 2 wt% at ambient temperature. The preparation methods, structure characteristics, improvement methods of hydrogen storage performance, and attenuation mechanism are systematically summarized and discussed. The relationships between hydrogen storage properties and alloy compositions as well as phase structures are discussed emphatically. For large-scale applications on MH tanks, it is necessary to develop low-cost and high-performance V–Ti-based solid solution alloys with high reversible hydrogen storage capacity, good cyclic durability, and excellent activation performance.

## Highlights

- Hydrogen storage performance of V-Ti-based solid solution alloys is related to the elementary composition, phase structure, and homogeneity.
- Micro-strain accumulation is responsible for capacity degradation.
- Low-cost and high-performance V-Ti-based solid solution alloys with high reversible hydrogen storage capacity, good cyclic durability, and excellent activation performance should be developed.

## Graphical abstract

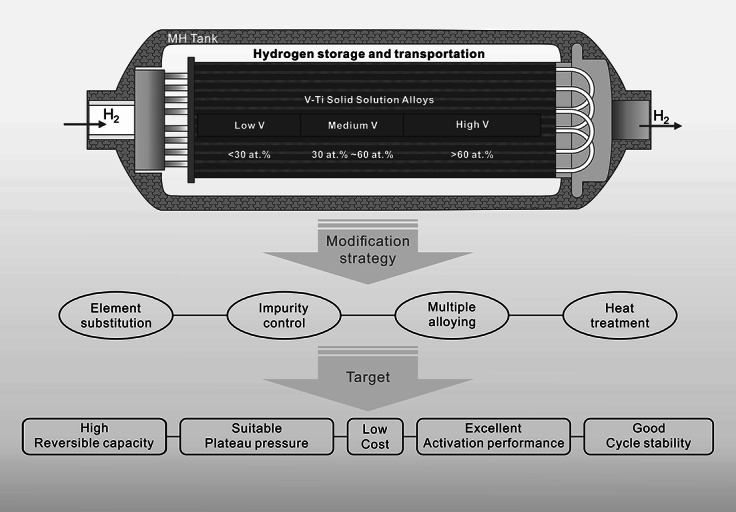

## Introduction

**Fig. 1:**
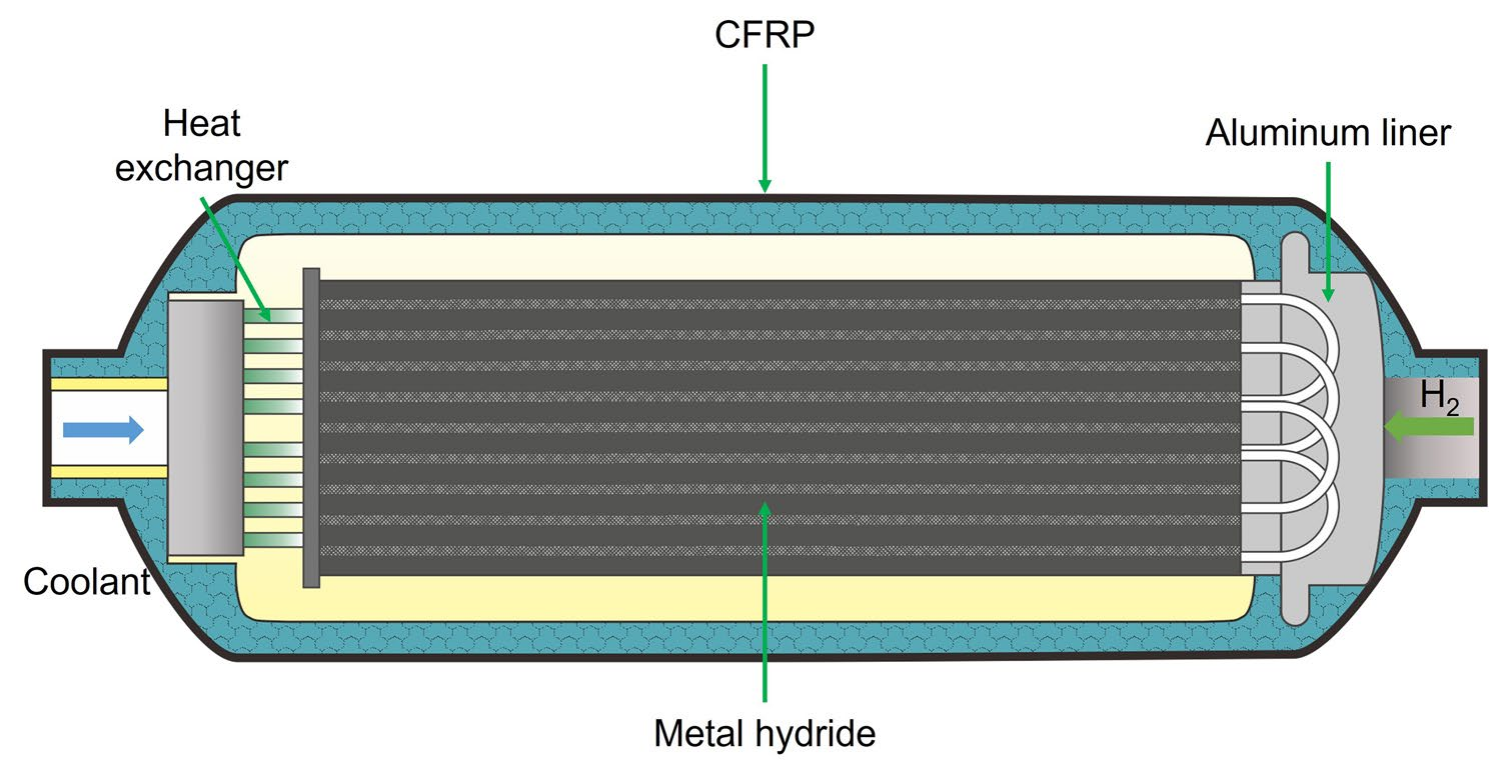
A schematic view of the MH tank

Solid-state hydrogen storage technology has received widespread attention because of the application of hydrogen energy in full swing and the requirement of high volumetric and gravimetric density candidates [1–3]. For on-board hydrogen storage, the vehicle-mounted hydrogen storage device is a reservoir that supplies hydrogen to fuel cells, which also requires a suitable hydrogen absorption/desorption rate at ambient temperature [4], as shown in Fig. 1. Consequently, acceptable economic cost, long cycle life, and safety for complex working conditions are more necessary for on-board hydrogen storage devices. In view of these, solid-state hydrogen storage technology provides an option to store hydrogen under non-extreme conditions (e.g., avoid 70 MPa or − 253 °C) [5].

Traditional hydrogen storage alloys, such as AB_5_-type, AB-type, and AB_2_-type alloys, typically have hydrogen capacities of less than 2.0 wt% [6, 7]. Despite the magnesium-based alloys having a higher hydrogen capacity (7.6 wt%), their high dehydrogenation temperature limits their application in on-board hydrogen storage devices [8–12]. Of course, by adding catalysts or applying nanoscale techniques, the dehydrogenation temperature can be effectively reduced while maintaining a hydrogen storage capacity above 6 wt% [13–15]. However, the actual dehydrogenation temperature is still difficult to match with the fuel cell module [16–18]. Metal borohydrides face a similar dilemma, and there is also the cost to consider [19]. Although the cost can be reduced by regenerating metal borohydrides from hydrolyzed products, further research is needed in terms of high dehydrogenation temperature, slow dehydrogenation kinetics and poor reversibility [20–22]. Reassuringly, V–Ti-based solid solution alloys exhibit a reversible hydrogen storage capacity of over 2.0 wt% at temperatures below 100 °C and are expected to be used on solid-state hydrogen storage devices [23, 24].

V–Ti-based solid solution alloys have been widely used and replaced metal V in many application areas because of their high hydrogen storage capacity, suitable hydrogen equilibrium pressure, and good anti-pulverization performance, such as solid-state hydrogen storage [25–27], Ni-MH battery [28–31], hydrogen separation [32–34], and hydrogen isotope effect [35]. However, since both V and Ti are hydrogen-stable metals, the resulting V–Ti alloy is also hydrogen-stable. For practical applications, it also encounters issues such as low effective hydrogen storage capacity, an uneven plateau area, and limited cyclic durability [23]. Therefore, multiple metallic elements are often simultaneously added to the alloy, forming a wide varieties of V–Ti-based hydrogen storage alloys with diverse properties.

This review summarizes the research progress of V–Ti-based solid solution alloys in recent years. The preparation methods, compositions, crystal structures, modification methods, and hydrogen storage properties of V–Ti-based solid solution alloys are fully discussed in this article.

## Preparation and Structural Characteristics of V–Ti-Based Solid Solution Alloys

**Fig. 2:**
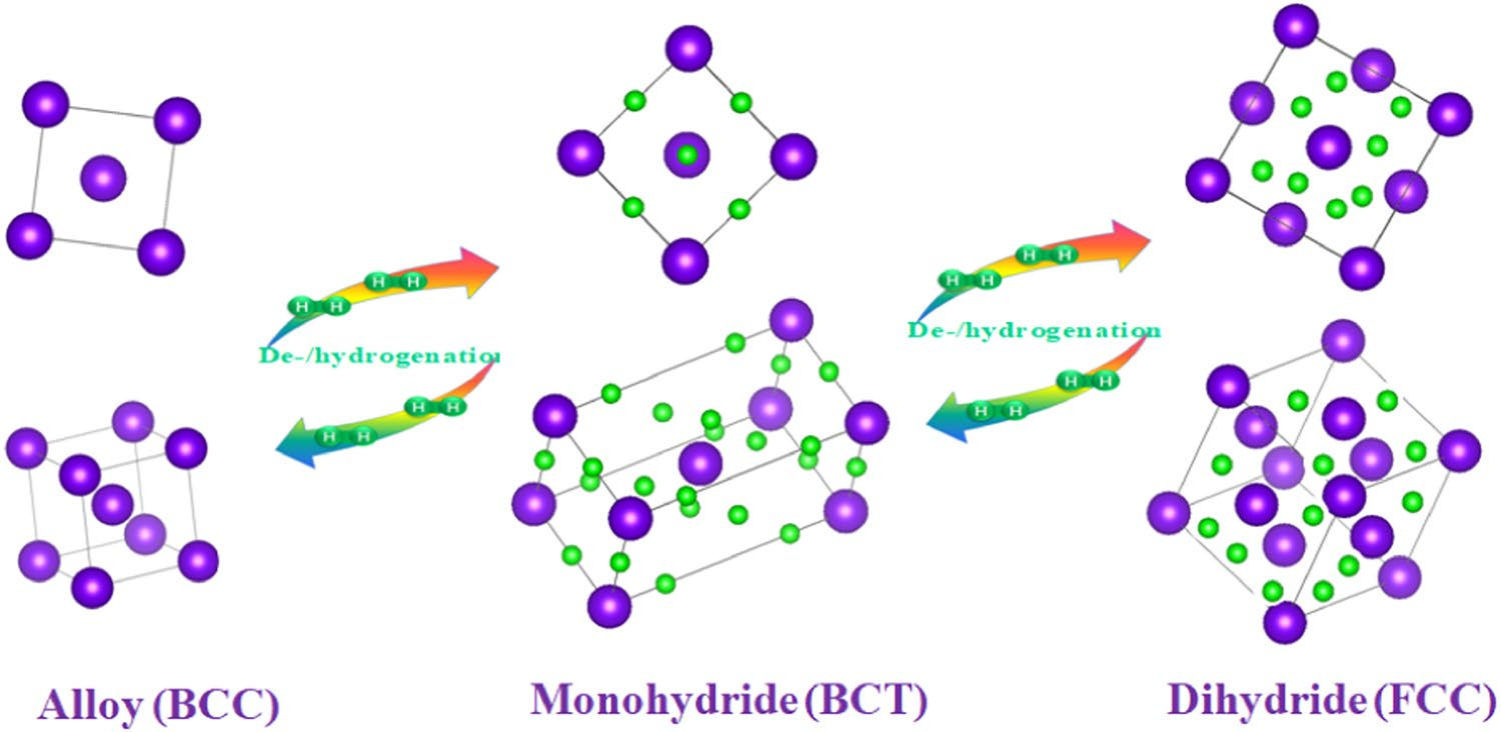
Schematic of the alloy crystal structure transformation upon de-/hydrogenation [12]. Copyright 2021 Elsevier

The metal V and Ti can be mutually soluble in any proportion, forming an infinite solid solution with a nearly 4 wt% hydrogen storage capacity. Since Akiba et al. [36] proposed the concept of “Laves phase-related BCC solid solution,” researchers have paid more attention to the V–Ti-based solid solution alloys. Generally, the V–Ti-based solid solution alloys change from the BCC phase (*α* phase) to the BCT phase (*β* phase) in the process of hydrogenation, and the relatively stable monohydride is formed at this stage. After further hydrogen absorption, the BCT phase converts to the FCC phase (*γ* phase), forming the dihydride. The dehydrogenation pathway of V–Ti-based solid solution alloys can be simply described as FCC(*γ*) → BCT(*β*) → BCC(*α*). Both hydrides have a wide range of non-stoichiometric ratios [37]. Therefore, although V–Ti-based solid solution alloys have a high theoretical hydrogen storage capacity of nearly 4 wt%, the actual effective hydrogen storage capacity is only about 2.6 wt% [24]. In fact, only the hydrogen storage capacity involved in phase transformation between the FCC phase and BCT phase is reversible, and the stable BCT phase generally cannot be dehydrogenated at near room temperature. And neutron diffraction results also show that the crystal structure of Ti_38_V_30_Cr_14_Mn_18_ alloy during the hydrogenation process transforms from the initial BCC structure to the BCT structure and then converts to FCC structure, in which the deuterium atoms occupy the octahedral sites of the BCC phase and tetrahedral sites of the BCT and FCC phases [38]. Therefore, the hydrogen absorption and desorption processes of V–Ti-based solid solution alloys can be summarized in Fig. 2 [12].

The preparation of alloys is related to the homogeneity of composition and phase, which is ultimately reflected in the hydrogen storage properties. For V–Ti-based solid solution alloys, in addition to ensuring the uniformity of the alloy, the content of impurities should also be strictly controlled, especially the oxygen content. Further, many of the elements involved in the V–Ti-based solid solution alloys, such as Ti, V, Cr, and Mn, are relatively active elements, which are easy to combine with oxygen to form metal oxides. Therefore, it is of practically significant to develop a suitable preparation process for the fabrication of high-quality and high-performance V–Ti-based solid solution alloys. Several methods, such as arc melting [39–44], vacuum magnetic levitation induction melting [45–48], powder sintering [49, 50], aluminothermy process [51, 52], ball milling [26, 53–59], suction casting [60, 61], floating zone melting [24, 62], rapid solidification [63] and laser engineered net shaping [64], have been used to prepare V–Ti-based solid solution alloys. In addition, heat treatment is also often used in combination with the above preparation methods, which will be detailed below.

Arc melting is the most common method for preparing V–Ti-based solid solution alloys, which is always in conjunction with a water-cooled copper crucible under an argon atmosphere [65]. Aoki et al. [66] prepared Ti_12_Cr_23_V_65_ and Ti_12_Cr_23_V_64_Fe_1_ alloys with single-phase BCC phase by arc melting and the BCC phase of the alloys converted to *γ* phase with an FCC crystal structure and *β* phase with a BCT crystal structure during de/hydrogenation, respectively. Yoo et al. [67] obtained Ti_0.32_Cr_0.33_V_0.25_Mn_0.1_ alloy by arc melting under Ar atmosphere with a BCC phase, while the Laves phase appeared with an Mn concentration of 10 at%. Suction casting is beneficial to avoid the formation of the TiCr_2_ phase, so it is easier to obtain single-phase BCC Ti–Cr–V alloys. For example, the as-cast (Ti_0.46_Cr_0.54_)_95_V_5_ alloy after arc melting consists of TiCr_2_ phase and BCC phase, while the alloy prepared by suction casting is a single BCC phase [60]. Compared with the as-cast alloy, grain sizes of the rapid solidification alloys were apparently refined and the phase abundance of the BCC phase for Ti_0.9_V_0.1_ alloy also increased [63].

Powder sintering is considered a suitable option that can be used for the manufacture of V–Ti-based solid solution alloys. Mao et al. [50] synthesized low-cost (FeV_80_)_48_Ti_26_Cr_26_ alloy using hydride powder sintering method and the sintered (FeV_80_)_48_Ti_26_Cr_26_ alloy could achieve the hydrogen storage properties comparable to the melted sample with Ti compensation. It is worth noting that a cheaper FeV_80_ alloy was used as the raw material and a reversible dehydrogenation capacity of 2.0 wt% was obtained at room temperature.

Ball milling has also been attempted to prepare V–Ti-based solid solution alloys. Cauceglia et al. [59] prepared the Ti–V–Ni alloy by ball milling, but unfortunately, the reversible hydrogen storage capacity of the alloy was only 1 wt%. Wang et al. [55] reported that the maximum hydrogen storage capacity of the Ti_0.37_V_0.38_Mn_0.25_ alloy prepared by mechanical alloying was 1.76 wt%, which was far lower than the capacity of the samples prepared by arc melting (3.62 wt%). The reason is that the samples prepared by arc melting have better crystal structures or crystallinity, which can provide more sites for the absorption of hydrogen atoms. In another study, the hydrogen storage capacity of the alloy decreased with the increase of ball milling time [58]. Therefore, ball milling changes the microstructure of the alloys and reduces grain size while increasing lattice strain and grain boundaries, and can also cause surface contamination [58]. The modification of the alloy prepared by arc melting using ball milling technology can significantly improve the kinetic properties of the alloy samples. The TiZrFeMnCrV alloy prepared by this method has ultra-fast hydrogen absorption kinetics, which can absorb 1.80 wt% hydrogen at a mild condition of 303 K, and the capacity is stable at about 1.76 wt% after 50 cycles, and the material is considered suitable for hydrogen storage in low-power fuel cells [68]. Zadorozhnyy et al. [69] compared the properties of single-phase BCC structured alloys prepared by arc melting (AM), electron beam melting with pendant drop melt extraction (EBM-PDME) as well as mechanical alloying (MA), and concluded that the alloys prepared by the AM and EBM-PDME methods had a hydrogen absorption of 1.6 wt% when fully transformed from the BCC phase to the FCC phase, in contrast to the MA alloy, which underwent partial amorphization and had a hydrogen storage capacity of less than 0.9 wt%. The authors attributed this discrepancy to the different equilibrium states of the alloys resulting from the different preparation processes. The high-temperature synthesis methods (AM and EBM-PDME) allow the metal atoms to occupy stable positions during the transition from the liquid phase to the solid solution, whereas mechanical alloying leads to the formation of highly disordered imperfect crystal structures that make them more susceptible to amorphization.

Like most high melting point alloys, V–Ti-based solid solution alloys can also be prepared by aluminothermic reduction, and the reaction is shown in Eq. (1). The high heat generated during the reaction melts the raw materials, and the product is separated from the Al_2_O_3_ slag after cooling. Recently, a V_82_–Ti_41_–Cr_73_–Al_0.08_ alloy was prepared by the aluminothermic method followed by vacuum arc melting [51]. This method is simple and inexpensive, but residual aluminum and other impurities will also affect the hydrogen storage properties of the alloy.

1 $${\text{M}}_{x} {\text{O}}_{y} {\text{ + Al}} \to {\text{M + Al}}_{{2}} {\text{O}}_{{3}}$$

The homogeneity of composition and phase significantly impacts the hydrogen storage performance of the alloy. Even after heat treatment, the fluctuation in vanadium concentration between regions with higher and lower vanadium content typically reaches up to 5%. Itoh and Arashima et al. [24, 62], prepared more homogeneous Ti–Cr–V alloys by floating zone melting and obtained higher reversible hydrogen storage capacity than that of the heat-treated alloys.

In general, researchers have been relentlessly striving to prepare alloys with uniform composition, controllable phase structure, and relatively low cost.

## Hydrogen Storage Properties of V–Ti-Based Solid Solution Alloys

The V–Ti-based solid solution alloys, as the main materials of the metal hydride tank to supply hydrogen to the fuel cells, need to have the following properties: (1) high reversible volumetric and gravimetric hydrogen capacity at low temperature; (2) suitable plateau pressure; (3) smaller slope factor; (4) good cyclic durability; (5) acceptable economic cost [70, 71]. In general, the hydrogen storage properties of V–Ti-based solid solution alloys are inseparable from their compositions and phase structures and have been extensively studied over the past few decades. Table 1 exhibits the reversible dehydrogenation capacities of some V–Ti-based hydrogen storage alloys at specific temperatures and the corresponding desorption plateau pressures. Based on information from some references, the roles of various elements in the V–Ti-based solid solution alloys are summarized and shown in Table 2.

**Table 1:** Hydrogen storage properties of various V–Ti-based solid solution alloys

	Temperature (K)	*P*_des_ (MPa)	Reversible desorption capacity (wt%)	$\Delta$*S* (J mol^−1^ K^−1^)	$\Delta$*H* (kJ mol^−1^)	Refs.
Ti_27_Cr_27_V_40_Fe_6_	338	–	2.25	151.56	51.33	[12]
Ti_25_V_40_Cr_35_	303	0.41	1.56	–	–	[42]
75V–Ti–15Cr–1Fe–1Al	298	0.5	2.26	–	–	[39]
Ti_24_V_40_Cr_34_Fe_2_	303	1.30	0.44	–	–	[42]
Ti_33_V_37_Mn_30_	363	0.322	1.82	–	33	[40]
Ti_33_V_37_Mn_29.4_Ce_0.6_	363	0.124	1.98	–	43	[40]
Ti_16_Cr_34_V_50_	293	–	2.24	–	–	[43]
Ti_16_Cr_34_V_49_Fe_1_	293	–	1.91	–	–	[43]
Ti_16_Cr_30_V_50_Nb_4_	303	–	2.21	–	–	[43]
Ti_25_Cr_25_V_25_Nb_5_	303	–	2.23	–	–	[43]
Ti_16_Zr_5_Cr_22_V_55_Fe_2_	298		1.42	–	–	[46]
(FeV_80_)_48_Ti_30_Cr_26_	298	0.2	2.0	–	–	[50]
V_40_Ti_26_Cr_6_Fe_8_	298	–	1.97	–	–	[49]
(VFe)_60_(TiCrCo)_40_	298	0.248	2.1	–	–	[64]
(VFe)_60_(TiCrCo)_40_Zr_0.5_	298	0.189	2.0	–	–	[64]
(Ti_0.32_Cr_0.43_V_0.25_) + 2 wt%La	293	–	2.23	–	–	[65]
Ti_12_Cr_23_V_65_	295	–	2.5	–	–	[66]
Ti_12_Cr_23_V_65_Fe_1_	273	–	2.42	–	–	[66]
TiCr_1.2_(FeV)_0.4_	318	–	1.36	–	–	[72]
V_30_Ti_32_Cr_32_Fe_6_	373	–	2.56	–223.6	–40.8	[73]
75 at%V–5 at%Ti–Cr	273	0.35	2.3	–	–	[70]
Ti_10_V_77_Cr_6_Fe_6_Zr	333	0.75	1.82	–	–	[74]
Ti_27.25_Cr_28.05_V_37.25_Fe_7.45_Ce_1.0_	343	–	2.25	–126.91	–41.97	[75]
Ti_32_Cr_46_V_22_Ce_0.4_	298	–	2.0	118	34.3	[76]
(V_30_Ti_35_Cr_2_Fe_10_)_97.5_Si_2.5_	298	0.22	1.22	–	–	[77]
(V_30_Ti_35_Cr_2_Fe_10_)_99.68_Si_0.32_	298	0.078	1.96	–	–	[77]
V_60_Ti_22.4_Cr_5.6_Fe_12_	298	0.062	2.12	–	–	[78]
Ti–28V–15Mn–10Cr	353	–	2.45	–	–	[79]
Ti_25_Cr_50_V_25_	273	–	2.30	–	–	[80]
Ti_25_Cr_49_V_25_Fe_1_	273	–	1.96	–	–	[80]
Ti_25_Cr_49_V_25_Nb_1_	273	–	2.38	–	–	[80]
Ti–25V–10Cr–25Mn	353	0.21	2.44	–	–	[81]
Ti–10Cr–18Mn–27V–5Fe	333	2.42	2.0	–	–	[82]
60 at%V–15 at%Ti–25 at%Cr	353	0.0101	2.62	–	–	[83]
Ti_25_Cr_50_V_25_	298	0.4	2.4	–	–	[84]
Ti_25_Cr_50_V_20_Mo_5_	298	2.3	2.4	–	–	[84]
V_35_Ti_35_Mn_30_	303	0.054	1.18	–	–	[85]
Ti_0.5_V_0.5_Mn	260	–	1.9	–	–26.8	[86]
Ti_1.5_Mo_0.5_CrV	298	–	2.23	–	49.14	[87]
V_0.60_5Ti_0.20_Cr_0.12_Mn_0.075_	303	0.31	1.92	–	–	[88]
(Ti_0.267_Cr_0.333_V_0.40_)_93_Fe_7_Ce_1.1_	298	–	2.05	–	–	[89]
V_40_Ti_23_Mn_37_	303	0.05	1.41	–	34.88	[90]
Ti_19_Hf_4_V_40_Mn_35_Cr_2_	293	–	1.58	–	–	[91]
Ti_19_Hf_4_V_40_Mn_37_	293		1.95	–	47.39	[92]
V_48_Fe_12_Ti_15_Cr_25_	295	1.01	1.66	123.55	30.12	[93]
V_48_Fe_12_Ti_14_Cr_25_Al_1_	295	0.92	1.46	113.24	28.02	[93]

**Table 2:** Properties of individual elements for V–Ti-based solid solution alloys

Elements	Primary functions	Refs.
V	Increase the BCC phase proportion Improve cyclic durability Increase hydrogen desorption plateau pressure Modulate hysteresis and slope of PCT curves	[24, 70] [90]
Nb	Increase the effective hydrogen storage capacity Improve the cyclic durability	[43, 80]
La/Ce	Improve the flatness of plateau Decrease slope factor Increase the effective hydrogen storage capacity Improve the activation property Suppress the influence of impurities Homogenize the composition and microstructure Decrease hydrogen desorption plateau pressure	[40, 65] [75, 76] [89, 94] [95, 96] [97]
Zr	Improved activation performance Improve the cyclic durability Suppress the Ti-containing phase separation Decrease the hydrogen ab/desorption capacities Decrease hydrogen desorption plateau pressure Decrease hysteresis and slope factor	[41, 64] [98, 99] [100]
Cr	Decrease the unit cell Increase the incubation period Slower hydrogen absorption kinetics Decrease hydrogen desorption plateau pressure Decrease slope factor	[33, 81]
Fe	Decrease the cell unit Increase the desorption plateau pressure Improve the activation property Improve the cyclic durability Decrease the hydrogen storage capacities Reduce the hysteresis	[42, 43, 66, 80, 82, 101]
Mn	Increase the plateau pressure Improve the activation property Increase the effective hydrogen storage capacity	[85, 88, 102]
Mo	Increase the desorption plateau pressure Improve the cyclic durability Weak stability of dihydride Reduce the hysteresis	[84, 87]
Si	Decrease the lattice parameter Increase the plateau pressure Improve the activation property Decrease the hydrogen ab/desorption capacities	[77, 103]
Al	Increase the lattice parameters of the BCC phase Increase the hydrogen desorption plateau pressure Decrease the hydrogen storage capacities Decrease the desorption kinetics Inhibit the formation of γ phase (dihydride) Increase the plateau hysteresis	[51, 93] [104] [105] [106]
B	Decrease the hydrogen storage capacities Increase the plateau pressure	[61]
O	Decrease the hydrogen storage capacities	[107]

### Classification of V–Ti-Based Solid Solution Alloys

According to the V content in the alloys, V–Ti solid solution alloys can be divided into high V (> 60 at% V), medium V (30–60 at% V), and low-V (< 30 at%) alloys. For low-V alloys, they are mostly composed of BCC, C14, or C15 phases. For medium and high V alloys, the BCC phase becomes the main phase with increasing V content. Of course, very few CeO_2_ and Ti–rich phases appear occasionally, depending on the composition and melting method. In general, low-V alloys tend to have low hydrogen storage capacity due to the presence of more C14 or C15 phases, but on the other hand, such alloys tend to have better activation performance. Besides higher hydrogen storage capacity, alloys with higher V content have better cycle stability due to the smaller bulk elastic modulus. From the initial binary V–Ti alloys to ternary, quaternary, and even quintuple alloys, a great deal of effort has been carried out to alter the hydrogen storage properties of these alloys.

### Composition Modification

Elemental substitution is a commonly used method to prepare V–Ti-based solid solution alloys with varied properties. These added elements can be broadly categorized into two types, hydrogen-stable metals and hydrogen-liable metals. It has been found that these substitutions not only reduce costs but also alter the hydrogen storage properties.

#### Hydrogen Stable Metal Substitution

The V–Ti-based solid solution alloys with high V content tend to have higher reversible hydrogen storage capacity such as 75V–Ti–15Cr–1Al–1Fe and 75 at%V-5 at%Ti–Cr [39, 70, 83]. Tsukahara et al. [83] obtained a 60 at% V-15 at% Ti-25 at% Cr alloy with a reversible hydrogen storage capacity of up to 2.62 wt% by adjusting the element content in the V–Ti–Cr alloy, demonstrating the potential of this alloy as a hydrogen carrier to supply hydrogen to fuel cells. It is generally believed that high V content can effectively improve the cyclic durability of V–Ti hydrogen storage alloys [24, 108]. Itoh et al. [24] investigated the durability of the highly homogenized Ti_8_Cr_12_V_80_ by floating zone melting and found that the desorption capacity of high V content Ti_8_Cr_12_V_80_ alloy only decayed by 1.4% after 500 cycles, which was smaller than for the Ti_24_Cr_36_V_40_ alloy. However, the price of metal vanadium is the most expensive among V–Ti-based solid solution alloys, so it is also meaningful to reduce the metal V content of the alloy on the basis of not affecting the performance of hydrogen storage alloys. Wang et al. [60] reported that the low-V suction-cast alloy rod (Ti_0.46_Cr_0.54_)_95_V_5_ had a maximum hydrogen uptake of 3.14 wt% at 333 K, but the actual reversible hydrogen storage capacity was only about 2 wt%. Furthermore, the (Ti_y_Cr_1-*y*_)_95_V_5_ (*y* = 0.38–0.54) alloys have a relatively high reversible hydrogen storage capacity and a flat plateau only when the Ti/Cr ratio is close to the CN_14_ cluster [Ti_7_Cr_8_]. Furthermore, the incorporation of vanadium plays a crucial role in modulating both the plateau slope and hysteresis characteristics of hydrogen storage alloys [70, 90].

**Fig. 3:**
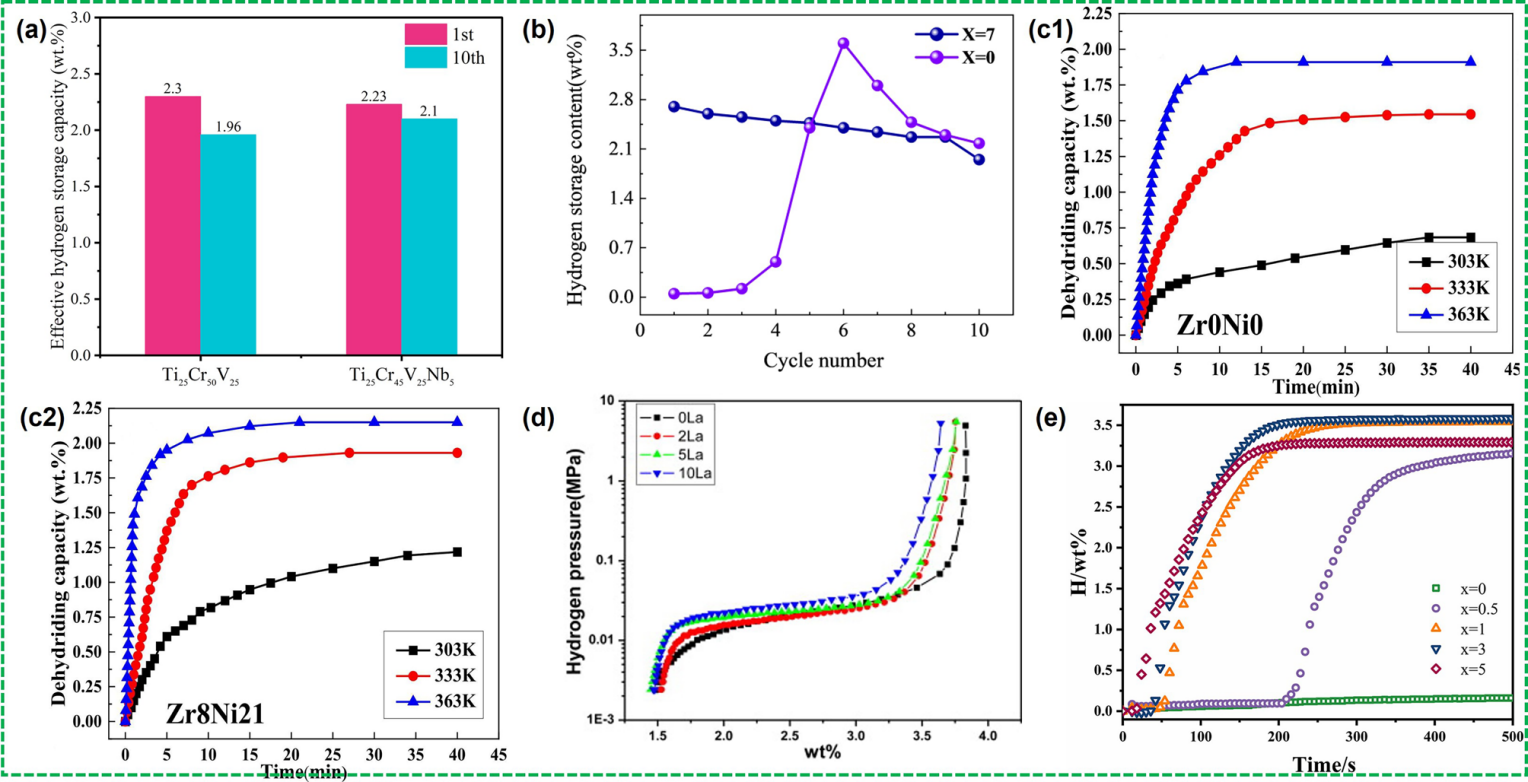
**a** Effective hydrogen storage capacity of Ti_25_Cr_50_V_25_ and Ti_25_Cr_45_V_25_Nb_5_ after 1st and 10th cycle. Reproduced with permission from Ref. [43]. Copyright 2013 Elsevier **b** Hydrogen capacities of Ti_50−*x*_V_25_Cr_25_Zr_*x*_ (*x* = 0 and 7) alloys over 10 cycles at 303 K. Reproduced with permission from Ref. [113]. Kinetics curves of hydrogen desorption of Ti_37_V_40_Mn_23_ + 10 wt% Zr_*x*_Ni_*y*_ alloys: **c1** Zr_0_Ni_0_ and **c2** Zr_8_Ni_21_. Reproduced with permission from Ref. [115]. Copyright 2024 Elsevier **d** Desorption P–C isotherms at 293 K for (Ti_0.32_Cr_0.43_V_0.25_) + *x* wt% La (*x* = 0, 2, 5, 10). Reproduced with permission from Ref. [65]. Copyright 2024 Elsevier **e** First hydrogen absorption kinetics of the Ti_28_Cr_26_V_35_Fe_6_Mo_5_ + *x* wt% Y (*x* = 0, 0.5, 1, 3, 5) alloys. Reproduced with permission from Ref. [120]. Copyright 2024 Elsevier

As an element with relatively strong hydrogen affinity, the Nb element can improve the cyclic durability without losing the effective hydrogen storage capacity of the V–Ti-based solid solution [80]. Towata et al. [43] compared the effect of Nb substitution on the hydrogen storage properties of V–Ti–Cr alloys. In the case of low-V Ti_25_Cr_45_V_25_Nb_5_ alloy, the first and 10th hydrogen storage capacities of the alloy were 2.23 and 2.10 wt%, while the 1st and 10th capacities of Ti_25_Cr_50_V_25_ were 2.30 and 1.96 wt%, respectively, indicating that the cycle stability of the sample after Nb substitution was improved without sacrificing the effective hydrogen storage capacity (Fig. 3a). The same is true for low-V Ti_25_Cr_45_V_25_Nb_5_ alloy. However, high Nb content, such as TiCrV_0.45_Nb_0.45_ alloy, can also lead to a significant decrease in desorption capacity [109]. Silva et al. [110] investigated in detail the hydrogen absorption and dehydrogenation properties of Nb-doped (TiVNb)_85_Cr_15_ alloy and found that the hydrogenation process went through the steps as alloy $\leftrightarrow$ BCC solid solution $\leftrightarrow$ BCC intermediate hydride $\leftrightarrow$ FCC dihydride, while the hydrogen absorption at lower hydrogen pressures reached 2 H/M. They also found that the H atoms were randomly distributed at the interstitial positions of the BCC and FCC structures rather than in a locally ordered state.

Zr substitution has been found to improve the hydrogen storage properties of V–Ti-based solid solution alloys [41, 98–100, 111, 112]. Suitable Zr substitution can improve the activation performance and reduce the lag loss, as well as increase the effective hydrogen storage capacity of the Ti_0.85_VFe_0.15_ alloy. Through a comparative analysis of the PCTs for Ti_0.85_VFe_0.15_ and Ti_0.85_V_0.95_Fe_0.15_Zr_0.05_, it was observed that the Zr-substituted system exhibited reduced hysteresis and a more uniform desorption plateau, which is essential for long-term stability [99]. For TiCrV alloy, Zr substitution led to a decrease in hydrogen absorption due to the formation of the ZrCr_2_ Laves phase. However, the cyclic hydrogen absorption performance was improved due to the inhibition of TiH_2_ phase separation [98]. The investigation further confirmed the improvement on the activation property and cyclic durability for Zr substitution [111]. The phase composition of (VFe)_60_(TiCrCo)_40_ alloy with Zr substitution comprises mainly a BCC main phase, C14 Laves phase, and CeO_2_ phase. The C14 phase obviously contributed to the improvement of the activation performance, and the addition of Zr reduced the strain accumulation during the hydrogen ab/desorption cycle. With the increment of Zr addition, the cyclic durability also improved accordingly, but the hydrogen storage capacity also decreased. In a recent study by Wang et al., the activation of the Ti_50_V_50_Cr_25_ alloy was greatly improved to reach 90% of the max hydrogen absorption capacity in just 230 s (Fig. 3b) by the addition of 7 wt% Zr, [113]. Zr can be used to refine V–Ti solid solution alloys with nano-eutectic structures. Zhang et al. [114] prepared Ti_40_V_60−*x*_Zr_*x*_ (*x* = 20, 25, 30) hydrogen storage alloys with nano-eutectic structures by arc melting, in which the hydrogen absorption of Ti_40_V_35_Zr_25_ alloy can reach 2.4 wt% in 10 min at 473 K with 1 MPa H_2_. Owing to the large difference in atomic size between V and Zr, the alloy generates large elastic energy, which leads to the formation of a stable multiphase eutectic structure that provides a high-density phase interface and generates a "synergistic effect," providing active sites and paths for H_2_ dissociation and diffusion, and thus promotes the improvement of the hydrogenation kinetics and hydrogen storage capacity, but the reversible hydrogen storage capacity (1.077 wt%) is unsatisfactory due to stable hydride formation. Liu et al. [115] added 10 wt% Zr_8_Ni_21_ alloy to Ti_37_V_40_Mn_23_ alloy by arc melting. Compared with the Ti_37_V_40_Mn_23_ alloy without ZrNi added, the dehydrogenation kinetics of the prepared Ti_37_V_40_Mn_23_ + 10 wt% Zr_8_Ni_21_ alloy at 303 K was significantly improved (Fig. 3c). It could release 2.15 wt% of hydrogen at 363 K and the dehydrogenation activation energy of the Ti_37_V_40_Mn_23_ + 10 wt% Zr_8_Ni_21_ alloy decreased to 55.20 kJ mol^−1^. Kamble et al. [116] compared the effect of introducing Zr through different methods on the hydrogen storage properties of TiCrV alloys and found that the introduction of 4 wt% Zr into Ti_52_V_12_Cr_36_ alloy by arc melting significantly improved the kinetic properties of the alloy wih a hydrogen storage capacity of up to 3.6 wt%, whereas the kinetic properties deteriorated when Zr was introduced through ball milling, and the authors attributed this phenomenon to the fact that ball milling only dispersed Zr on the surface of the particles that cannot form a ternary phase with Ti, Cr, and V.

It is recognized that the addition of rare earth elements can effectively improve the activation properties of alloys [65, 89, 95–97]. Singh et al. [65, 95] found that the La element has the best effect by comparing the effects of La, Y, Ce, and LaNi_5_ on the hydrogen storage properties of V–Ti–Cr alloys. Although only 91% of the BCC phase involved in the dehydrogenation process, the Ti_0.32_Cr_0.43_V_0.25_ + 5 wt% La composite exhibited a desorption capacity of 2.31 wt% at 293 K and a flatter plateau after La substitution, as shown in Fig. 3d. It should be noted that there is no significant reduction in effective hydrogen storage capacity, and the slope of the plateau was markedly diminished, which is positive for practical applications. In addition, Ce substitution with a suitable content can effectually homogenize the BCC phase, which reduces the oxygen concentration and suppresses the Ti-containing second phase separation, facilitating a more gradual plateau [89]. Due to the effective properties of deoxygenation, Ce element is often added to remove impurity elements in FeV master alloys. Liu et al. [76] studied the effect of Ce addition on hydrogen storage performance of Ti_32_Cr_46_V_22_ alloy, and the results showed that Ce addition effectively improved the desorption plateau pressure and effective hydrogen storage capacity of the V_32_Cr_46_V_22_ alloy. Notably, the slope of the plateau experienced a significant reduction. Chen et al. [40]. found that the BBC phase abundance of Ti–V–Mn alloys increased with the increase of Ce content, thus achieving a higher reversible hydrogen storage capacity. And Ce or CeH_2.51_, rather than CeO_2_, played a key role in the activation process, which had a more important effect than the C14  Laves phase in V–Ti-based solid solution alloys [40, 97].

At present, there are few studies on Sc and Y substitution. In Kwon et al.’s report [117], moderate Sc substitution achieved a higher hydrogen storage capacity than the original Ti_0.32_Cr_0.43_V_0.25_ alloy, but the actual effective desorption capacity was reduced. In a separate study, however, it seemed that it had little effect on the improvement of the performance of 40Ti–40V–10Cr–10Mn alloys [118]. Excessive Sc substitution can result in the inability to embed into the BCC structure, while the generated ScH_2_ can lead to a decrease in the effective desorption capacity at 373 K. By comparing the four V_35_Ti_35_Cr_10_Fe_10_M_10_ (M = Mn, Co, Sc, or Ni) HEA alloys, Sc can indeed accelerate hydrogenation kinetics to a certain extent [119]. Chen et al. [120] introduced 1 wt% Y into the Ti_28_Cr_26_V_35_Fe_6_Mo_5_ alloy and achieved better activation performance. After Y doping, the incubation period was reduced to 204 s (Y: 0.5 wt%) and below 50 s (Y: > 1 wt%), respectively (Fig. 3e). On the one hand, yttrium formed YH_2_ during the hydrogenation process, providing a channel for H diffusion during activation. On the other hand, the introduction of yttrium reduced the pulverization of the alloy. Liang et al. [121] introduced Y element into Ti–V–Mn alloy to prepare Ti_0.9_V_1.1_Mn_0.9_Y_0.1_ tetrameric alloy, which could absorb 3.54 wt% hydrogen in 3 min at room temperature and release 3.05 wt% hydrogen when dehydrogenated at 473 K until 1 bar, and the high reversible hydrogen storage capacity can be attributed to the coexistence of Y phase and C14 Laves phase.

In order to improve the weight hydrogen storage density of the alloy, Mariana et al. [122] prepared a Mg_35_Al_15_Ti_25_V_10_Zn_15_ alloy by mechanical alloying under an argon atmosphere with Mg as the doping element, and the addition of Zn element was favorable for the formation of solid solution and avoiding the segregation of Mg. The prepared samples consisted of a BCC phase and a certain amount of unmixed Mg and could absorb 2.5 wt% hydrogen at 648 K and 4 MPa hydrogen pressure.

A small amount of Pd can also effectively improve the activation performance of Ti_33_V_33_Cr_34_ alloy and improve its cycle stability [123]. However, Pd is even more expensive than metal V, making it difficult for practical application.

#### Hydrogen Labile Metal Substitution

V–Ti–Cr system is one of the most widely explored V-based alloys and the introduction of Cr improves the cyclic durability and powder resistance of V–Ti alloys without affecting their reversible hydrogen storage capacity, and the incorporation of Cr also facilitates a decrease in the plateau slope [81]. However, the long incubation time and the high temperature and high-pressure conditions required in the first activation process increase the manufacturing cost of the V–Ti–Cr alloys [33, 81]. The reason for the difficulty in activation is still inconclusive, but the oxide layer on the surface of the alloy is recognized as one of the culprits. To improve the activation properties of the V–Ti–Cr alloys, researchers have carried out plenty of research. Yu et al. [124] mixed quenched Ti–28V–15Mn–10Cr alloy with 10 wt% LaNi_3.55_Co_0.75_Mn_0.4_Al_0.3_ alloy by ball milling and found that the activation performance of the obtained composite was greatly improved, which was because the layer of LaNi_3.55_Co_0.75_Mn_0.4_Al_0.3_ on the surface of the alloy can effectively improve the diffusion and absorption of H. However, the effect of LaNi_3.55_Co_0.75_Mn_0.4_Al_0.3_ addition on other hydrogen storage properties was not given in this study.

For the V–Ti–Cr alloys, the effective hydrogen storage capacity and hydrogen desorption pressure of the alloys are mainly related to the Ti/Cr ratio and the V content [60, 70, 125–127]. When the Ti/Cr ratio is constant, the main phase of the alloys changes from Ti–Cr compounds to the BCC phase with the increase of V content. At the same time, the hydrogen storage performance of the alloys is greatly improved, but the activation performance becomes worse. Because H atoms diffuse more easily in Ti–Cr compounds with larger lattice constant, and the increase of the abundance of BCC phase raises H atom diffusion resistance [126]. The lattice parameters of the V–Ti–Cr alloys increase with the Ti/Cr ratio, which is not only related to the effective hydrogen storage capacity of the alloys but also associated with the cycle durability [33, 127]. Lin et al. [33] compared the cycling performance of V–Ti–Cr alloys with different Ti/Cr ratios, and the results show that the cycling durability of Ti_0.8_Cr_1.2_V was better than TiCrV at various temperatures. Goshome et al. [128] investigated the performance of V_40_TiCr and V_70_TiCr as metal hydride hydrogen compressors and regulated the hydrogen absorption and desorption plateau of the alloys by varying the value of Ti/Cr, which in turn affects the hydrogen compression performance and cycling stability of the alloys. In order to reveal the occupancy of H atoms in V–Ti–Cr alloys, Sakaki et al. [129] used synchrotron radiation and neutron total scattering experiments to investigate the structure of the dihydride phases formed by V_10_Ti_35_Cr_55_, V_50_Ti_20_Cr_30_, and V_80_Ti_8_Cr_12_ alloys, and the results show that the dihydride phases have an FCC structure in the whole and in the local area, and the crystal structure is CaF_2_ structure, the interatomic distance between hydrogen and Cr is shorter than that with V, and the interatomic distance with Ti is longer than that with V, indicating that the hydrogen atoms do not occupy the centers of the tetrahedra but move toward the Cr atoms and away from the Ti atoms.

**Fig. 4:**
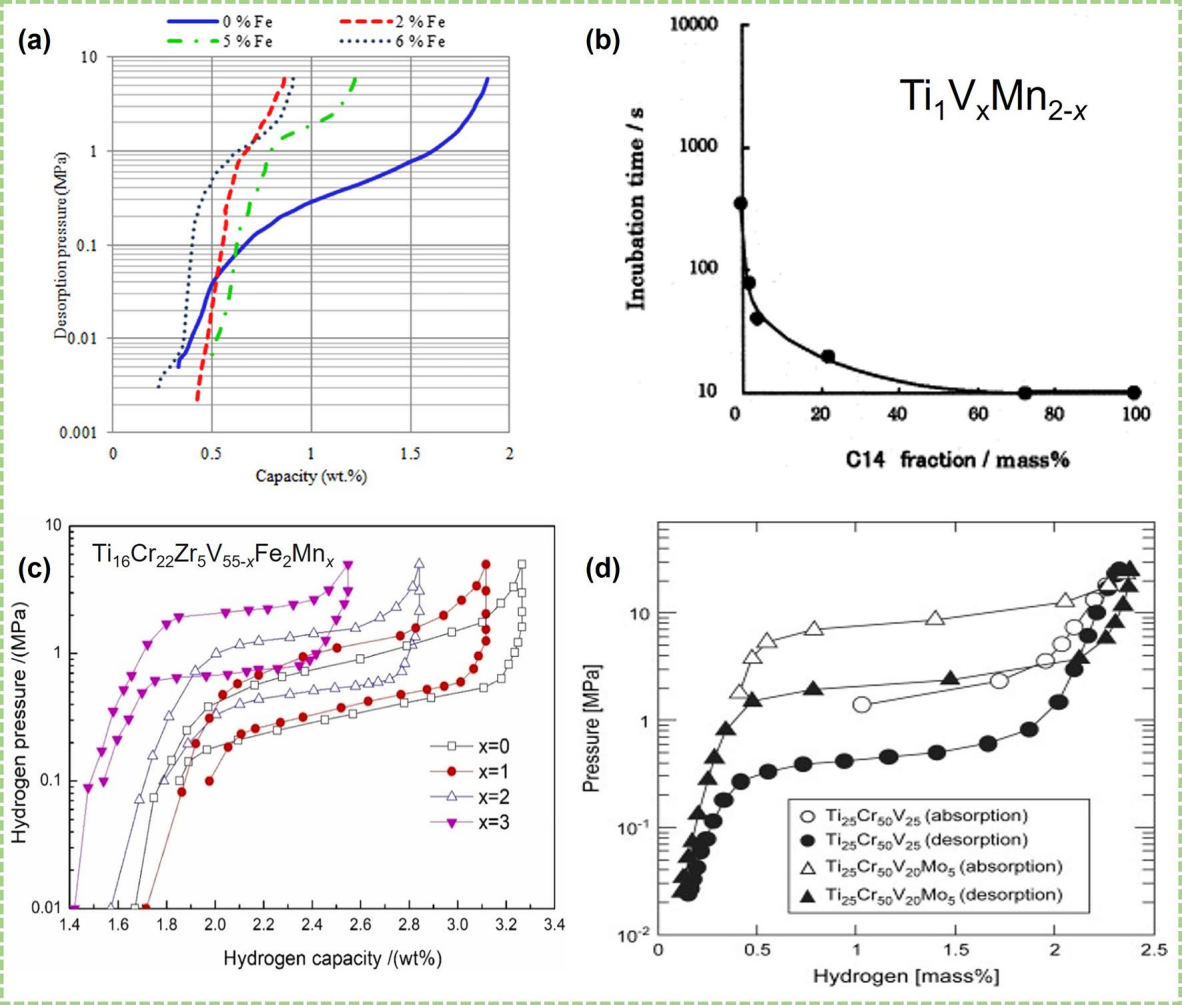
**a** Effect of Fe on H desorption in Ti_25−0.5*x*_V_40_Cr_35−0.5*x*_Fe_*x*_ (*x* = 0, 2, 5, 6 at%) alloys. Reproduced with permission from Ref. [42]. Copyright 2016 Elsevier **b** Relation between C14 phase fraction and incubation time. Reproduced with permission from Ref. [102]. Copyright 2002 Elsevier **c** PCT curves of Ti_16_Cr_22_Zr_5_V_55−*x*_Fe_2_Mn_*x*_ (*x* = 0–3) samples at 298 K. Reproduced with permission from Ref. [133]. Copyright 2024 Elsevier **d** PC isotherms of TiCrV and TiCrVMo. Reproduced with permission from Ref. [84]. Copyright 2009 Elsevier

Fe element is also often introduced into the V–Ti-based solid solution alloys to improve the reversible hydrogen storage capacity of the alloys. For V–Ti–Cr alloys, Fe substitution will occupy Ti and Cr sites, resulting in a decrease in unit cell parameters and an increase in plateau pressure. As shown in Fig. 4a, Abdul and Chown prepared a series of Fe-doped Ti_35–0.5*x*_V_40_Cr_25–0.5*x*_Fe_*x*_ alloy and all Fe-doped alloys exhibited higher desorption plateau [42]. But the side effect of Fe substitution is also obvious, that is, the plateau becomes steeper, and the hydrogen storage capacity decreases [42, 72, 101]. The effect of Fe substitution in the Ti–V–Mn system is different from that in the Ti–V–Cr system. Santos et al. [130] prepared a Ti–Mn–(FeV) solid solution alloy by replacing V with FeV alloy, which is equivalent to replacing vanadium with Fe in the Ti–V–Mn system. And the reversible hydrogen storage capacity of Ti–Mn–(FeV) alloy can be significantly increased despite the adverse effects of impurities in FeV alloy, which can be attributed to the fact that the desorption plateau pressure of V–Ti–Mn alloys is relatively low and the higher plateau pressure after Fe substitution is more favorable for hydrogen release. The Fe element also can improve the activation performance and cycle durability of V–Ti-based solid solution alloys [46, 66, 80, 82]. Hang et al. [46] obtained the Ti_16_Zr_5_Cr_22_V_55_Fe_2_ alloy by partial substitution of Fe for V with BCC main phase and C14 secondary phase, which has good activation performance and exhibits an effective hydrogen storage capacity of 1.42 wt%. Aoki and Towata et al. [43, 66] focused on the effect of trace Fe on the cycle performance of Ti–Cr–V alloy and found that 1 at% Fe can significantly improve the cycle performance of the alloy. It is true that 1 at% Fe substitution slightly reduced the hydrogen storage capacity of the alloy from 2.50 to 2.42 wt%, but the capacity retention rate of Ti_12_Cr_23_V_64_Fe_1_ alloy after 100 cycles is 97%, while that of Ti_12_Cr_23_V_65_ alloy is only 88% [66]. So, it seems worthwhile to sacrifice a tiny hydrogen storage capacity for a huge improvement in cycle durability. But for alloys with lower V content such as Ti_16_Cr_34_V_50_ alloy, both the maximum hydrogen storage capacity and the effective hydrogen storage capacity will be greatly reduced after only adding 1 at% Fe [43]. If the amount of iron added is too high, even the high V alloy Ti_16_V_60_Cr_24_, its effective hydrogen storage capacity will be significantly reduced [131].

Similar to V–Ti–Cr alloys, the phase structure of V–Ti–Mn alloy formed by Mn substitution is also related to the V content and the Ti/Mn ratio [85, 90, 102, 132]. Matsuda et al. [132] studied the effect of V content on the phase structure of V–Ti–Mn alloy and found that when the V content is greater than 40%, the alloy will only contain BCC phase; and when the V content is less than 26.7%, more than 90% of the alloy in the alloy phase is C14 phase. When the V content is constant, the proportion of the C14 phase in the alloys increases with the decrease of the Ti/Mn ratio. As mentioned in most literature, Mn substitution promotes the phase structure transformation of alloys from the BCC phase to the C14 phase. And the highly active C14 phase has good activation properties and can greatly shorten the incubation time of V–Ti–Mn alloys, as shown in Fig. 4b [102]. However, it will also lead to a significant decrease on the hydrogen storage capacity of the alloys because the C14 phase has a lower hydrogen storage capacity than the BCC phase [85, 88, 90, 102]. The lattice parameter of the BCC phase increases with the increase of the Ti/Mn ratio, which leads to an increase in the hydrogen absorption rate of the alloy, but the formed hydride is also more stable, making it difficult to dehydrogenate. Taghizadeh et al. [48] regulated the ratio of BCC phase and C14 phase in the alloys by remelting technique, which led to a decrease of BCC phase volume fraction, and the slope and hysteresis coefficient of the PCT curves decreased with increasing number of remelts. Recent studies by Hang et al. [133] have also shown that tiny Mn introduction can improve the flatness of the desorption plateau and plateau pressure of the Ti_16_Zr_5_Cr_22_V_55−*x*_Fe_2_Mn_*x*_ (*x* = 0, 1, 2, 3) alloys (Fig. 4b). It is a pity that the reversible hydrogen storage capacity of V–Ti–Mn alloys reported in the literature is mostly less than 2.0 wt% at ambient temperature (< 100 °C) [40, 85, 86, 134].

Mo can improve the plateau desorption pressure, the effective hydrogen storage capacity, and the cyclic durability of V–Ti-based solid solution alloys [84, 87]. Matsunaga et al. [84] studied the effect of Mo on the hydrogen storage properties of Ti_25_Cr_50_V_25_ alloy and found that the dehydrogenation plateau pressure of Ti_25_Cr_50_V_20_Mo_5_ alloy at 298 K increased from 0.4 to 2.3 MPa after 5 at% Mo substitution, and the effective hydrogen storage capacity is still the original 2.4 wt% (Fig. 4d). Similar results were obtained by Hu et al. [87], the Ti_1.5_Mo_0.5_CrV alloy exhibited a reversible hydrogen storage capacity of 2.33 wt% at 298 K, and the cycle durability was also greatly improved. This can be attributed to the decrease of dihydrides stability as well as the enthalpy change value (∆*H*), and the hysteresis of dehydrogenation process is also significantly reduced. Recently, Lin et al. [135] studied the effect of Mo introduction on the cycling properties of low-V alloy TiCr_1.2_(V–Fe)_0.6_, and they believed that the introduction of Mo can effectively inhibit the formation of the second phase during the cycling process.

In addition, V–Ti-based solid solution alloys substituted with other metal elements, such as Hf [91, 92], and Rh [136] have also been extensively studied to improve the hydrogen storage performance of the alloys. The highest desorption capacity of 1.88 wt% at 293 K was achieved for the Hf substituted-Ti_23_V_40_Mn_37_ alloy. In addition, V–Ti–Ni alloys are mainly used in hydrogen separation/purification and Ni-MH batteries, which will not be repeated here.

At present, V–Ti-based solid solution alloys have been developed in the direction of multi-alloying, such as quaternary and quinary alloys, to meet the needs of different application scenarios with high effective hydrogen storage capacity, suitable plateau pressure, and lower dehydrogenation temperature, etc. Yoo et al. [67] studied the effect of the simultaneous substitution of Cr by Mn and Fe on the hydrogen storage properties of the Ti–Cr–V alloy. Not only the reversible hydrogen storage capacity of the prepared Ti_0.32_Cr_0.32_V_0.25_Fe_0.03_Mn_0.08_ alloy at 293 K was increased from 2.3 to 2.5 wt%, but also the desorption plateau pressure was improved, while Mn substitution alone almost made plateau pressure no significant elevation. Zhu et al. [137] also confirmed the synergistic effect of Nb, Fe, Co, Ni, and Mn elements in improving the hydrogen storage properties, and TiCrV_0.7_(Nb_0.2_Fe_0.2_Co_0.2_Ni_0.2_Mn_0.2_)_0.2_ alloy had excellent hydrogen storage properties. It is also important to note that the activation performance of the alloy is crucial for on-board hydrogen storage devices. Because the difficulty of activation determines whether the process can be achieved in the MH tank. Several studies focusing on activation properties have also been carried out [138, 139]. The reduction of alloy particle size would help to reduce the difficulty of alloy activation, but the introduction of the second phase had a more obvious effect on the improvement of alloy activation performance [138].

**Fig. 5:**
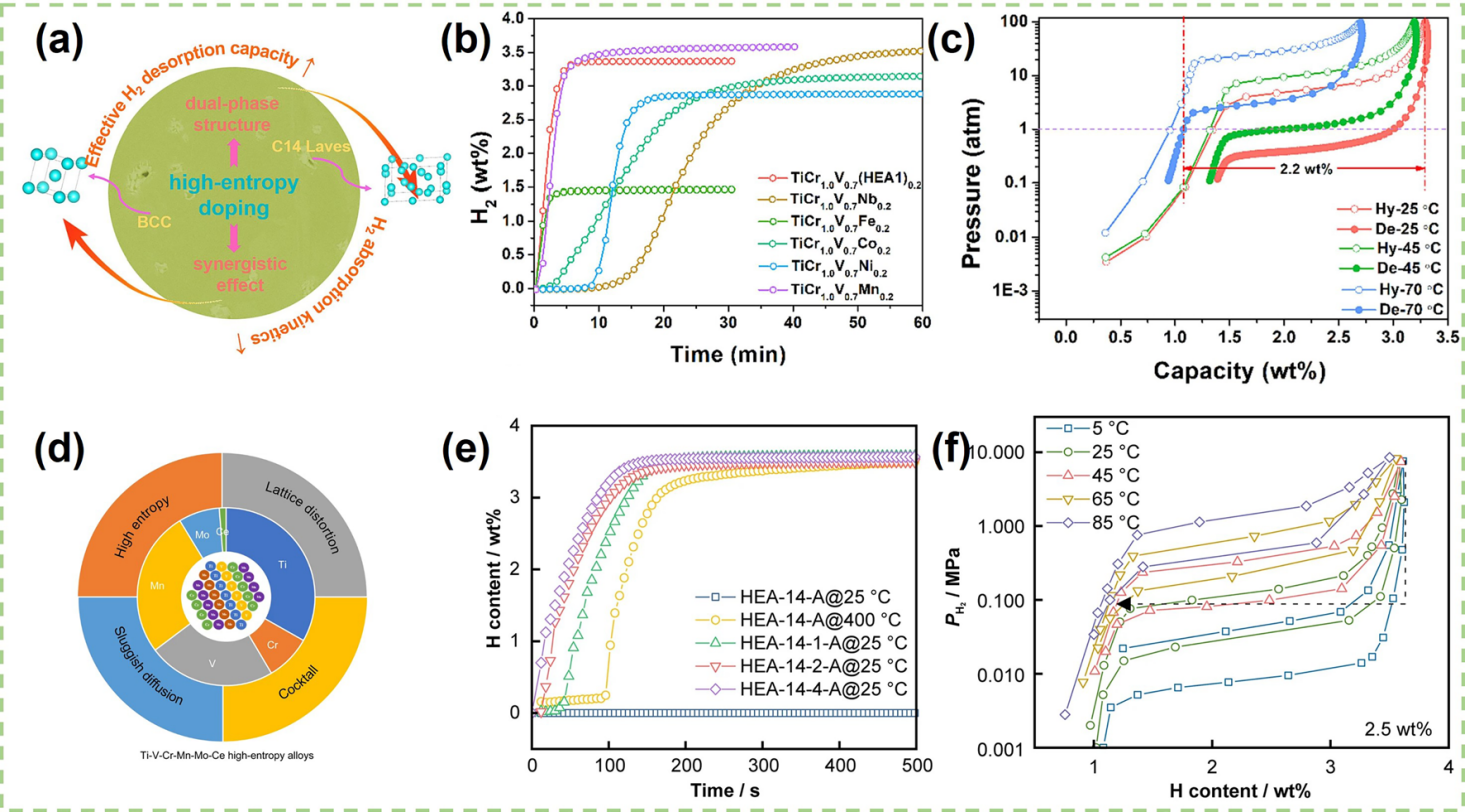
**a** Schematic diagram of compositionally complex (high-entropy) doping strategy. **b** Hydrogen absorption kinetics of different alloys at 25 °C and 3 MPa H_2_. **c** De-/hydrogenation PCT curves of heat-treated TiCr_1.0_V_0.7_(HEA1)_0.2_ alloy at different temperatures. **a**–**c** Reproduced with permission from Ref. [137]. Copyright 2023 Elsevier **d** Schematic diagram of Ti–V–Cr–Mn–Mo-Ce high-entropy alloys. **e** First hydrogen absorption kinetics of HEAs after activation at different temperatures. **f** PCT curves of HEA-14-1-A alloys at different temperatures. **d**–**f** Reproduced with permission from Ref. [152]. Copyright 2024 Springer Nature

Multi-component high-entropy alloys (HEAs) are the current research hotspot [68, 140, 141] and their unique crystal structures directly mean that they have hydrogen storage properties that are different from conventional alloys, and they can be precisely modified by appropriate design, including chemical composition, crystal structure, valence electron concentration or lattice distortion parameters, and so on. The entropy characteristics of high-entropy alloys diverge significantly from those of traditional alloys. The total mixing entropy encompasses configurational entropy ($\Delta S_{{{\text{mix}}}}^{{{\text{conf}}}}$), vibrational entropy ($\Delta S_{{{\text{mix}}}}^{{{\text{vib}}}}$), magnetic dipole entropy ($\Delta S_{{{\text{mix}}}}^{{{\text{mag}}}}$), and electronic randomness entropy ($\Delta S_{{{\text{mix}}}}^{{{\text{elec}}}}$). The mixing entropy is dominated by configurational entropy. Li et al. believe that there is a certain relationship between valence electron concentration and hydrogen storage capacity for V_35_Ti_35_Cr_10_Fe_10_M_10_ (*M* = Mn, Co, Sc, and Ni) alloys, and when valence electron concentration exceeded 5.17, hydrogen storage capacity began to decrease [119]. Ma et al. [142] prepared a TiVZrNbFe HEA by vacuum arc melting, which could absorb 1.60 wt% hydrogen at 323 K and 1 MPa for only 100 s. These results are similar to those of the TiZrFeMnCrV HEA [68] and have excellent kinetic properties. Recently, Serrano et al. [143] designed three HEAs with the alloy compositions of Ti_35_V_35_Nb_20_Cr_5_Mn_5_, Ti_32_V_32_Nb_18_Cr_9_Mn_9_, and Ti_27.5_V_27.5_Nb_20_Cr_12.5_Mn_12.5_ by using the CALPHAD method. These HEAs absorbed hydrogen to form FCC hydrides with hydrogen storage capacities of 2.47, 2.09, and 3.38 wt%, respectively, and possessed excellent activation properties. The introduction of Cr and Mn promoted the formation of the AB_2_-type Laves phase, which lowered the stability of the hydrides and facilitated the desorption of the hydrides. Balcerzak et al. [140] developed a low-crystallinity TiVFeCuNb HEA in accordance with the criteria established for single-phase high-entropy alloys ($\Delta S_{{{\text{mix}}}}^{{{\text{conf}}}}$ ≥ 1.61 *R*; − 15 kJ mol^−1^ <∆H_mix_< − 5 kJ mol^−1^; *δr* < 6.6%; Ω > 1.1, where *R* is a gas constant), and the test results showed that the alloy had excellent kinetic properties and could reach half of its hydrogen storage capacity within 20 s. Regrettably, the maximum hydrogen storage capacity of the alloy is only 0.6 wt%. In addition, HEA can also be used as dopants for V–Ti-based solid solution alloys to improve the hydrogen storage properties of the alloys. Zhu et al. [137] provided a composite (high-entropy) doping strategy to prepare a low-cost TiCrV_0.7_(Nb_0.2_Fe_0.2_Co_0.2_Ni_0.2_Mn_0.2_)_0.2_ alloy (Fig. 5a). Compared with with single element substitution, the TiCr_1.0_V_0.7_(HEA1)_0.2_ alloy displayed excellent activation properties (Fig. 5b). After heat treatment, the TiCr_1.0_V_0.7_(HEA1)_0.2_ alloy could release 2.21 wt% H_2_ cutting-off at 1 atm at 70 °C after fully absorbing hydrogen at lower temperature of 25 °C (Fig. 5c). Preparation of single-phase HEAs is an important task to improve reversiable hydrogen storage capacity [69, 144–151]. Montero et al. [149] prepared a single-phase Ti_0.30_V_0.25_Zr_0.10_Nb_0.25_Ta_0.10_ HEA with a hydrogen storage capacity of up to 2.5 wt%, which remained at 2.2 wt% after 10th hydrogen absorption and desorption cycles. Recently, Chen et al. doped 1 at% Ce into V–Ti–Cr–Mn–Mo alloy to form a BCC-type Ti–Cr–V–Mn–Mo–Ce HEA (Fig. 5d). This stratagy resulted in a shorter incubation period of 40 s and a significant decrease of ∆H from 46.89 to 17.96 kJ mol^−1^ for the alloy (Fig. 5e). Although the introduction of Ce element reduced the hydrogen desorption plateau, the HEA-14-1-A alloy still achieved an effective improvement of hydrogen desorption capacity from 1.10 to 2.50 wt% (Fig. 5f) [152]. Many researchers have sought to prepare single-phase HEAs based on thermodynamic modeling [153–156] and experimentally to verify the correctness of the theory. Rapid quenching of melt is considered to be an effective method for the preparation of single-phase HEAs [157].

### Heat Treatment

**Fig. 6:**
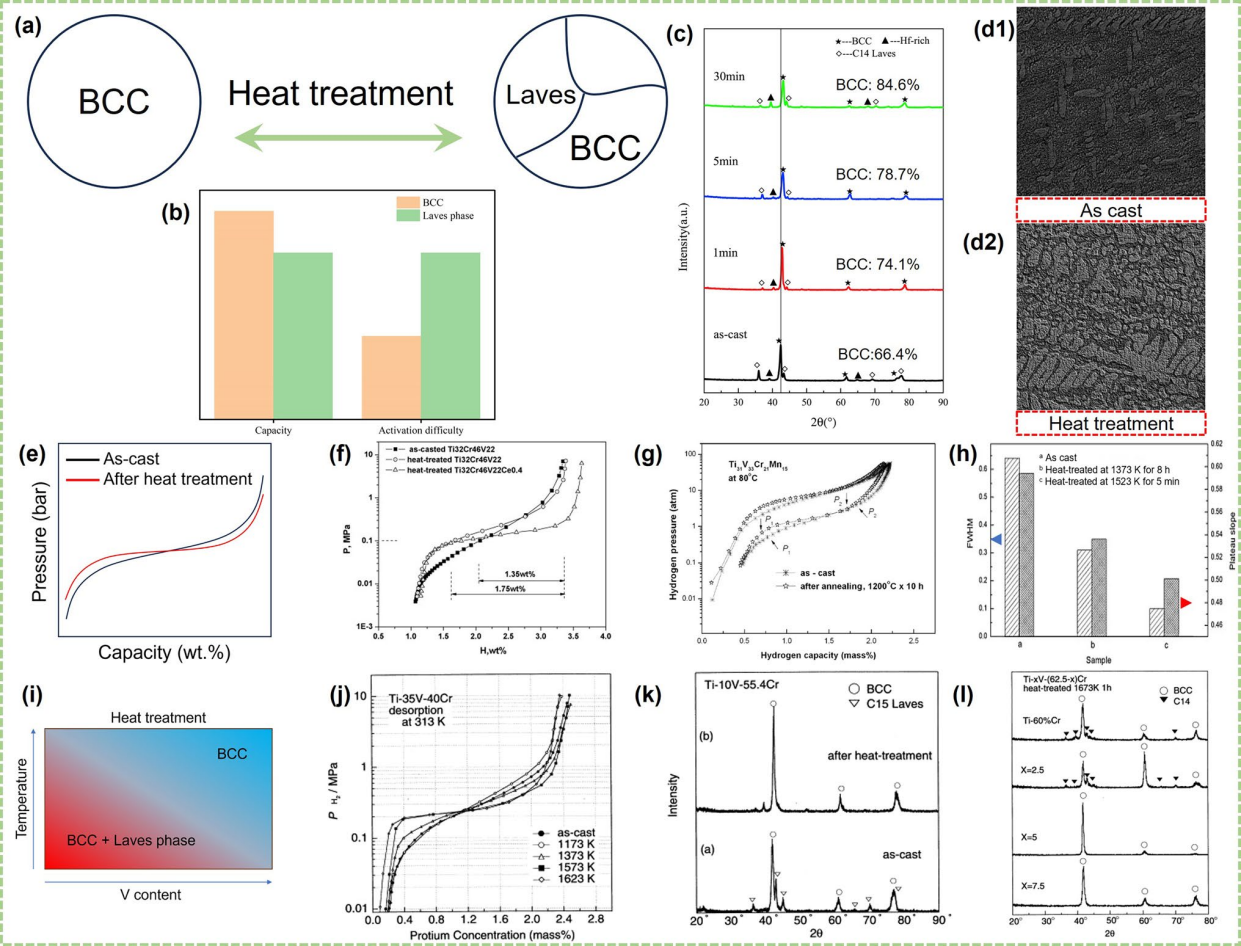
**a** Schematic diagram of phase transformation caused by heat treatment. **b** Comparison of hydrogen storage capacity and activation difficulty of BCC phase and Laves phase. **c** XRD patterns and corresponding BCC phase abundance of as-cast and heat-treated Ti_19_Hf_4_V_40_Mn_35_Cr_2_ alloys. Reproduced with permission from Ref. [165]. Copyright 2023 Elsevier Microstructure of Ti_42.75_Zr_27_Mn_20.25_V_10_ alloy: (**d1**) as-cast; (**d2**) after heat treatment at 900 °C. Reproduced with permission from Ref. [163]. Copyright 2021 Elsevier **e** Diagram of the effect of heat treatment on the PCT curves. **f** Dehydrogenation p–c isotherms of Ti_32_Cr_46_V_22_ and Ti_32_Cr_46_V_22_Ce_0.4_ alloys at 298 K. Reproduced with permission from Ref. [76]. Copyright 2009 Elsevier g PCI results for Ti_31_V_33_Cr_21_Mn_15_ alloy before and after heat treatment. Reproduced with permission from Ref. [159]. Copyright 2011 Taylor&Francis. **h** The relationship between FWHM and desorption plateau slope of the as-cast and heat-treated samples: **a** as-cast; **b** heat-treated at 1373 K for 8 h; **c** heat-treated at 1523 K for 5 min. Reproduced with permission from Ref. [74]. Copyright 2012 Elsevier **i** Diagram of the effect of heat treatment temperature and V content on phase composition for V–Ti–Cr alloys. **j** P–C–T curves at desorption process of Ti–35V–40Cr alloys with a single b.c.c. phase annealed at 1173–1623 K for 2 h. **k** P–C–T curves at desorption process of Ti–35V–40Cr alloys with a single b.c.c. phase annealed at 1173–1623 K for 2 h. **l** XRD patterns of the Ti–*x*V–Cr (*x* = 0–7.5) alloys after a heat treatment at 1673 K for 1 h. **j**–**l** Reproduced with permission from Ref. [161]. Copyright 2002 Elsevier

Heat treatment directly affects the hydrogen storage properties of the alloys due to the homogenization of the composition and the transformation to the structure that occurs during the process. Exactly, heat treatment in most cases means heat treating at a certain temperature for a period of time and then quenching in water in this review [74, 76, 84, 94, 158–160]. Table 3 summarizes the heat treatment conditions carried out for some V–Ti solid solution alloys and the corresponding phase structures. As shown in Fig. 6a, heat treatment may cause phase transformation, mainly between BCC and Laves phases. In general, the hydrogen storage capacity of BCC phase is higher than that of Laves phase, but the Laves phase is easier to activate than the BCC phase (Fig. 6b). Therefore, a satisfactory alloy always achieves a delicate balance between the two.

**Table 3:** Heat treatment conditions and corresponding phase structures of some V–Ti-based solid solution alloys

Alloys	Heat treatment		Phase composition	Refs.
	Temperature (K)	Time (min)		
Ti–35V–45Cr	1573	1	BCC + secondary phase	[161]
Ti–10V–55.4Cr	1573	1	BCC + C14 Laves	[161]
Ti–5V–57.5Cr	1673	60	BCC + C14 Laves	[161]
V_30_Ti_32_Cr_32_Fe_6_	1673	30	BCC	[73]
V_0.605_Ti_0.20_Cr_0.12_Mn_0.075_	1573	480	–	[88]
V_48_Fe_12_Ti_15_Cr_25_	1273	600	BCC + Ti–rich + TiFe	[162]
T_32_Cr_46_V_22_Ce_0.4_	1673	5	BCC + CeO_2_	[76]
V_35_Ti_20_Cr_45_	973	4320	BCC + C14 Laves	[158]
Ti_31_V_33_Cr_19_Mn_15_	1473	600	–	[159]
Ti_0.16_Zr_0.05_Cr_0.22_V_0.57_	1473	480	BCC	[160]
Ti_0.95_Zr_0.05_V_0.2_Mn_1.3_	1173	4320	BCC	[44]
Ti_42.75_Zr_27_Mn_20.25_V_10_	1173	1200	BCC + C14 Laves	[163]
Ti_10_V_77_Cr_6_Fe_6_Zr	1573	5	BCC + C14 Laves	[74]
Ti_25_Cr_50_V_20_Mo_5_	1473	120	BCC	[87]
Ti_0.32_Cr_0.39_V_0.25_Sc_0.04_	1523	480	BCC	[117]
(Ti_0.267_Cr_0.333_V_0.40_)_93_Fe_7_Ce_1.1_	1673	5	BCC + CeO_2_/Ce	[89]
TiCrVMo	1473	120	BCC	[84]
Ti_26.5_Cr_20_(V_0.45_Fe_0.085_)_100_Ce_0.5_	1673	5	BCC + secondary phase	[104]
Ti_24_Cr_17.5_V_50_Fe_8.5_Ce_1_	1673	5	BCC + CeO_2_	[94]
Ti_24_Cr_36_V_40_	1673	180	–	[164]
Ti_0.5_V_0.51_Mn	1233	360	BCC + FCC + C14 Laves	[134]
V_40_Ti_21.5_Cr_38.5_	1500	1440	BCC	[108]
Ti_25_Cr_45_V_25_Nb_5_	1600	10	BCC	[80]
V_60_Ti_22.4_Cr_5.6_Fe_12_	1673	30	BCC	[78]

On the one hand, heat treatment promoted the conversion of the second phase to the BCC phase and increased the effective dehydrogenation capacity of the alloys under certain conditions [73, 74, 76, 117, 160]. The as-cast V_30_Ti_32_Cr_32_Fe_6_ alloy with BCC and C14 Laves phases changed into a single BCC phase after heat treatment at 1673 K for 30 min, which contributed to the increase of hydrogen storage capacity from 2.1 to 2.35 wt% at 298 K [73]. Liu et al. [165] compared the BCC phase abundance of as-cast and heat-treated Ti_19_Hf_4_V_40_Mn_35_Cr_2_ alloys, and found that the BCC phase abundance of the heat-treated Ti_19_Hf_4_V_40_Mn_35_Cr_2_ alloys was higher than that of the as-cast alloy (Fig. 6c). Both the C14 phase and the ZrCr_2_ phase in the Ti_0.16_Zr_0.05_Cr_0.22_V_0.57_ alloy disappeared after heat treatment [160]. Hang et al. [74] systematically investigated the influence of different heat treatment conditions on microstructure and hydrogen storage properties of the Ti_10_V_77_Cr_6_Fe_6_Zr alloy and found that the phase abundance of BCC annealed at 1523 K for 5 min effectively increased and the lattice strain decreased, leading to the increase of actual reversible hydrogen storage capacity. But heat treatment sometimes can also lead to an increase in the phase abundance of the C14 phase. For example, the proportion of the C14 phase increased in V_35_Ti_20_Cr_45_ and Ti_42.75_Zr_27_Mn_20.25_V_10_ alloys after a heat treatment at 973 K for 72 h and 1173 K for 20 h respectively, which resulted in easier activation of the alloys [158, 163]. Figure 6d shows the microstructure of as-cast and heat-treated Ti_42.75_Zr_27_Mn_20.25_V_10_ alloys, which can be visually seen the increase of the Laves phases (C14 type). Luo et al. [162] also found that the effective hydrogen storage capacity of V_48_Fe_12_Ti_15_Cr_25_ alloy decreased after annealing although the hydrogen absorption and desorption kinetics were accelerated. Actually, the generation of the C14 phase after heat treatment improves the activation properties of the alloys. The reason for the two distinct phenomena may be that the BCC phase is stable at higher temperatures, whereas the Laves phase is not. High temperature and short time of heat treatment are more favorable to obtain BCC phase alloys.

On the other hand, heat treatment is shown to cause the plateau flatter by decreasing the lattice strain [73, 74, 76, 159, 160], as demnonstrated in Fig. 6e. Liu et al. [76] studied the effect of heat treatment on the plateau of BCC alloys and found the plateau was flatter than that of as-casted alloy after heat treatment. The Ti_32_Cr_46_V_22_ alloy heat-treated at 1673 K for 5 min could desorb 1.75 wt% H_2_ at 298 K and a pressure of 0.1 MPa, while the as-cast sample only released 1.35 wt% (Fig. 6f). There is no doubt that a flatter plateau is more conducive to hydrogen release. In the study conducted by Jeng et al. [159], the desorption plateau pressure of the Ti_31_V_33_Cr_21_Mn_15_ alloy increased from 1.43 to 2.33 atm after heat treatment at 1473 K for 10 h, and the slope factor decreased from 3.67 to 2.24 accordingly (Fig. 6g). The full width half maximum (FWHM) of the X-ray diffraction decreased, indicating that lattice strain was reduced after heat treatment, which was consistent with the change in the plateau slope factor [73, 76]. In addition, it can be seen from Fig. 6h that different heat treatment conditions have different effects on FWHM and the slope factor [74]. In brief, the change of effective hydrogen storage capacity of the alloys after heat treatment was influenced by the variation of phase structure and desorption plateau pressure.

Empirically, the temperature and duration of heat treatment need to be increased and prolonged respectively with the decrease of V content in the V–Ti–Cr alloys (Fig. 6i). Okada et al. [161] systematically investigated the effect of heat treatment on the phase structure and hydrogen storage performance of V–Ti–Cr alloys. For the Ti–35V–40Cr alloy, annealing above 1573 K was effective for flattening the plateau region and increasing the effective hydrogen storage capacity (Fig. 6j). For the Ti–10V–55.4Cr alloy, the C15 Laves phase could still be converted to BCC phase after annealing at 1573 K for 1 min (Fig. 6k). While the alloys with less than 10 at% V were more suitable to obtain more BCC phase at higher temperatures and longer periods of time (1673 K for 60 min), as shown in Fig. 6l. In other words, for the alloy with less V content, higher heat treatment temperature and longer duration time are needed to obtain more BCC main phase. Yu et al. [79] obtained a Ti–28V–15Mn–10Cr alloy with smaller grains quenched by a water-cooled rotating molybdenum disk, which induced the C14 phase to disappear and converted to the BCC phase during the component homogenization process. Under the condition of 353 K, the plateau of the quenched Ti–28V–15Mn–10Cr alloy was flatter than that of the as-cast sample, and the hydrogen storage capacity was also improved from 2.3 to 2.45 wt%. In addition, the prolongation of annealing time led to a further precipitation of the Ti–rich phase, and the Ti–35V–40Cr alloy annealed at 1573 K for 1 min showed about 2.6 wt% hydrogen storage capacity, but the capacity decreased significantly after extending the annealing time to 50 h. The quenching treatment sometimes had an adverse effect on the V–Ti-based hydrogen storage alloys. For example, the first activation process after quenching becasme more difficult than the as-cast sample [79].

### Degradation Mechanism

The degradation of capacity is affected by many factors, which can be divided into two categories, intrinsic factors and extrinsic factors. The intrinsic factors include element composition, particle size, micro-strain, dislocation density, and so on. Extrinsic factors mainly include impurity gas (O_2_, H_2_O, CO, etc.) and cyclic testing conditions. The attenuation of capacity is offen influenced by multiple factors superimposed on each other in practical applications.

**Fig. 7:**
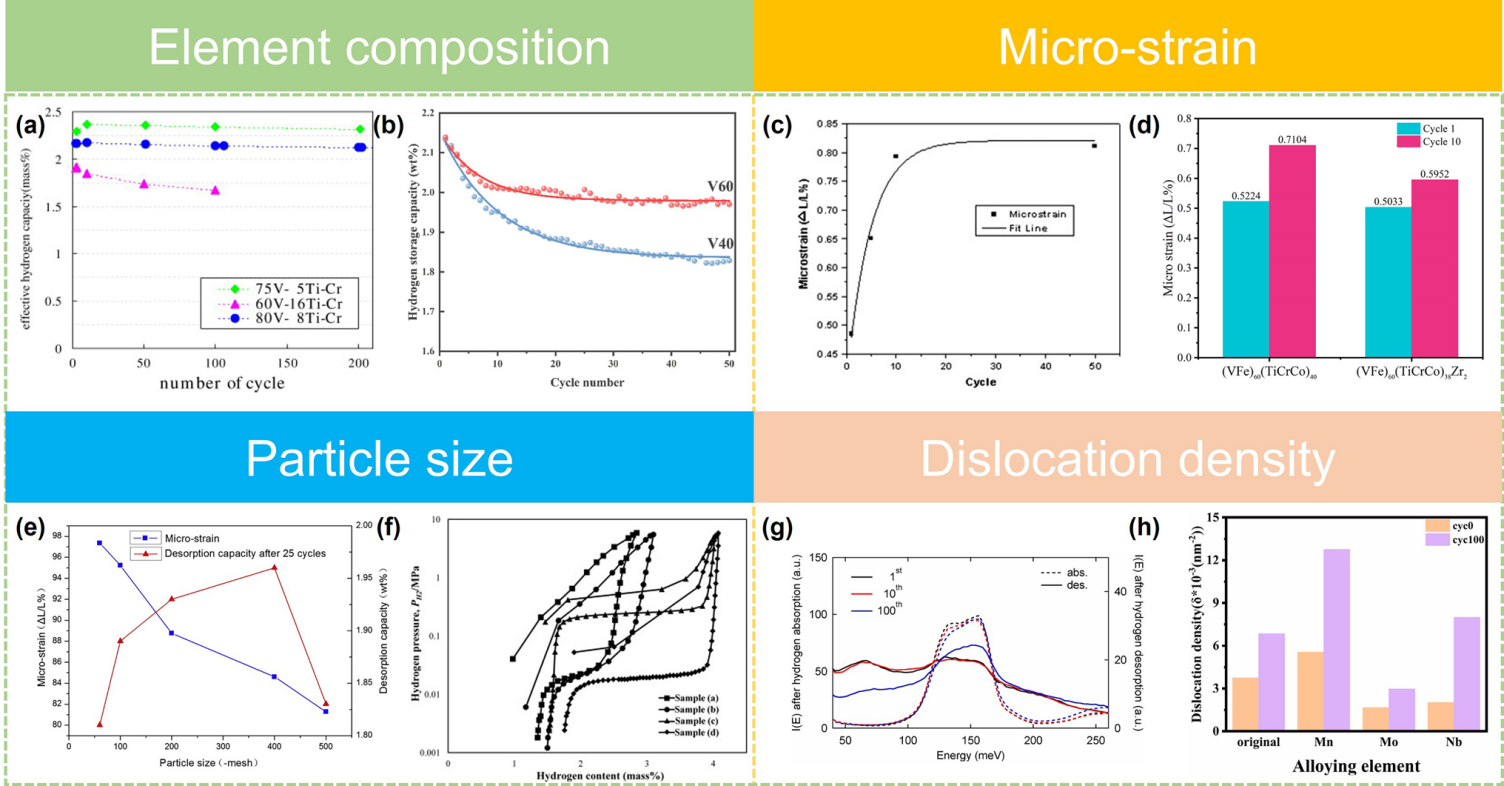
**a** Relationship between effective hydrogen capacity and cycle number of 75at%V–5at%Ti–Cr as-cast sample along with those of 60at%V–16at%Ti–Cr and 80at%V–8at%Ti–Cr. Reproduced with permission from Ref. [70]. Copyright 2010 Elsevier **b** Hydrogen storage capacities of the V40 and V60 alloys at 333 K during cycling. Reproduced with permission from Ref. [166]. Copyright 2023 Elsevier **c** Fitting of micro-strain of V_60_Ti_22.4_Cr_5.6_Fe_12_ monohydride during absorption/desorption cycles. Reproduced with permission from Ref. [78]. Copyright 2010 Elsevier **d** Micro-strain of the (VFe)_60_(TiCrCo)_40−*x*_Zr_*x*_ alloys (*x* = 0, 2) after different hydrogen absorption–desorption cycles. Reproduced with permission from Ref. [111]. Copyright 2016 Elsevier **e** The relationship patterns of desorption capacity and micro-strain of (VFe)_48_(TiCrMn)_52_ alloy with different particle sizes after the 25th cycle. Reproduced with permission from Ref. [167]. Copyright 2015 Elsevier **f** P–C isotherms of the exploded particles with **a** composition #1, **b** composition #2 and the mechanically crushed particles from ingot with **c** composition #2, **d** composition #3 measured at 293 K. Reproduced with permission from Ref. [168]. Copyright 2012 Elsevier **g** Inelastic neutron scattering intensities, I(E), after hydrogen absorption and desorption of V_0.10_Ti_0.36_Cr_0.54_. Reproduced with permission from Ref. [171]. Copyright 2024 Elsevier **h** Dislocation density of original alloy (TiCr_1.2_(V–Fe)_0.6_) and TiCr_1.1_M_0.1_(V–Fe)_0.6_ (*M* = Mn, Mo, Nb) alloys before hydrogenation and after cycling. Reproduced with permission from Ref. [135]. Copyright 2024 Elsevier

The element composition of an alloy directly affects its cyclic properties. Generally, the high V alloys have better cycle stability than the low-V alloys [108]. Selvaraj et al. [108] found that the low-V alloy would undergo disproportionation during the cycles and a small fraction of Ti and V would be precipitated and subsequently converted into corresponding hydrides, which may be one of the reasons for the poor cyclic durability of low-V alloys. Kuriiwa et al. [70] gave detailed data on the effects of V content and Ti/Cr ratio on the cyclic properties of the alloy. As shown in Fig. 7a, both 80V–8Ti–Cr and 75V-5Ti–Cr alloys showed good cycle stability compared with the 60V–16Ti–Cr alloy. In addition, Ti/Cr ratio played a key role to obtain higher effective hydrogen capacity as well as good capacity retention rate. Recently, Yang et al. [166] compared the microstructures and hydrogen storage performances of three V contents alloys (20%, 40%, and 60%). The V_60_Ti_19_Cr_19_Fe_2_ alloy (V60) achieved better cycle stability than the V_40_Ti_28.5_Cr_30.1_Fe_1.4_ (V40) alloy (Fig. 7b). The authors believed that the smaller bulk elastic modulus due to the higher V content effectively reduced strain accumulation and volume expansion.

The micro-strain accumulated during cycles will distort lattice interstices and cumber or even prohibit the entry of hydrogen atoms, leading to a significantly degraded hydrogen capacity [111, 164]. Wu et al. [78] studied the hydrogen storage capacity and micro-strain changes of the V_60_Ti_(21.4+*x*)_Cr_(6.6−*x*)_Fe_12_(0 ≤ *x* ≤ 3) alloys during hydrogen absorption and desorption cycles. The experimental results show that with the progress of hydrogen absorption and desorption, the reversible hydrogen storage capacity of the alloy was constantly attenuated. At the same time, its micro-strain was also increasing during ab/desorption cycles (Fig. 7c), and the macroscopic realization was a decrease in the desorption plateau pressure.

Zr substitution can reduce the micro-strain accumulation during the hydrogen ab/desorption cycles as well as Fe and Nb elements [66, 80, 111]. By comparing the changes of FWHM, Aoki et al. [66] found that Fe substitution inhibited the increase of lattice strain in the hydrogen ab/desorption cycles, thus greatly improving the cycle durability of Ti_12_Cr_23_V_64_Fe_1_ alloy, as shown in Fig. 7. A similar effect was also concluded in the work conducted by Yang et al. [111] which showed that the micro-strain increase of Zr-substituted (VFe)_60_(TiCrCo)_40_ alloy during hydrogen ab/desorption cycles was suppressed, and the capacity decay was only 4.5% after 10 cycles. Zr substitution, however, did not inhibit the growth of micro-srain in the alloy (Fig. 7d). There is no definitive explanation as to why the substitution of Fe, Nb, and Zr, etc. elements can reduce strain accumulation. In addition, annealing can effectively reduce dislocations in the Ti–Cr–V alloys but the dehydrogenation performance cannot be improved, because the dislocation accumulation mainly occurred in the hydrogen absorption process [164].

In addition, micro-strain is closely related to particle size. Luo et al. [167] investigated the decaying behaviors of the V_40_(TiCr)_51_Fe_8_Mn alloys with different particle sizes (60, 100, 200, 400, and 500 mesh) and found that the decay rate of dehydrogenation capacity decreased with the decreasing of alloy powder particle size (from 60 mesh down to 400 mesh). While the particle size was reduced to 500 mesh, the hydrogen storage properties of V_40_(TiCr)_51_Fe_8_Mn alloy deteriorated in all aspects (Fig. 7e). When the particle size of the alloy powder is blindly reduced, the hydrogen storage performance of the alloy will deteriorate. Kown et al. [168] found that the hydrogen storage capacity of the nano/micro-particle containing alloys decreased compared to that of the mechanically crushed particles from ingots (Fig. 7f). The reasons for this phenomenon have not yet been determined though there are several viewpoints focusing mainly in the following two points. One is that a smaller particle size makes the alloy powder easy to be poisoned by external factors, and the other is that an alloy powder with a smaller particle size increases the specific surface area. Therefore, the sites for H atom occupancy are reduced, resulting in a decrease in the hydrogen storage capacity [167, 168].

Dislocation density is closely related to micro-strain and particle size. Kim et al. [169] investigated the origin of the capacity degradation of V–Ti-based solid solution alloys during cycling by using the atomic pair distribution function (PDF) analysis, and the results showed that the large number of dislocations generated in the alloys during cycling is closely related to the capacity fading. Ball milling often introduces defects and strain in the material. Wu et al. [170] observed that the hydrogen storage capacity of the V_60_Ti_25_Cr_3_Fe_12_ alloy decreased with the prolongation of milling time. Correspondingly, the strain and dislocation density in the alloys gradually increased during ball milling and make insuffient improvement of cycle stability. Recently, Ikeda et al. revealed that the degradation of hydrogen storage capacity stems from the introduced dislocations during the ab/desorption cycle by inelastic neutron scattering. The intensity at different energy (meV) represents different H-occupied sites of octahedral sites and tetrahedral sites. After 100 cycles, the inelastic neutron scattering intensities curves changed with a decrease in hydrogen at the octahedral sites and a similar increase at the tetrahedral sites, which is different from the both hydrogen occupation of tetrahedral and octahedral sites during the early cycles (Fig. 7g). Li et al. [135] explored the effect of adding different metal elements (*M* = Mn, Mo, Nb) on the capacity decay of TiCr_1.1_M_0.1_(V–Fe)_0.6_ alloys, and they observed that the capacity retention rate of TiCr_1.1_Mo_0.1_(V–Fe)_0.6_ alloy was the highest after Mo subsitition. It is found that both the grain size and micro-strain of the TiCr_1.1_Mo_0.1_(V–Fe)_0.6_ alloys were increased after cycling, but the dislocation density of the alloy with Mo subsitition was the lowest after cycling (Fig. 7h), which might be the reason for its high capacity retention rate.

**Fig. 8:**
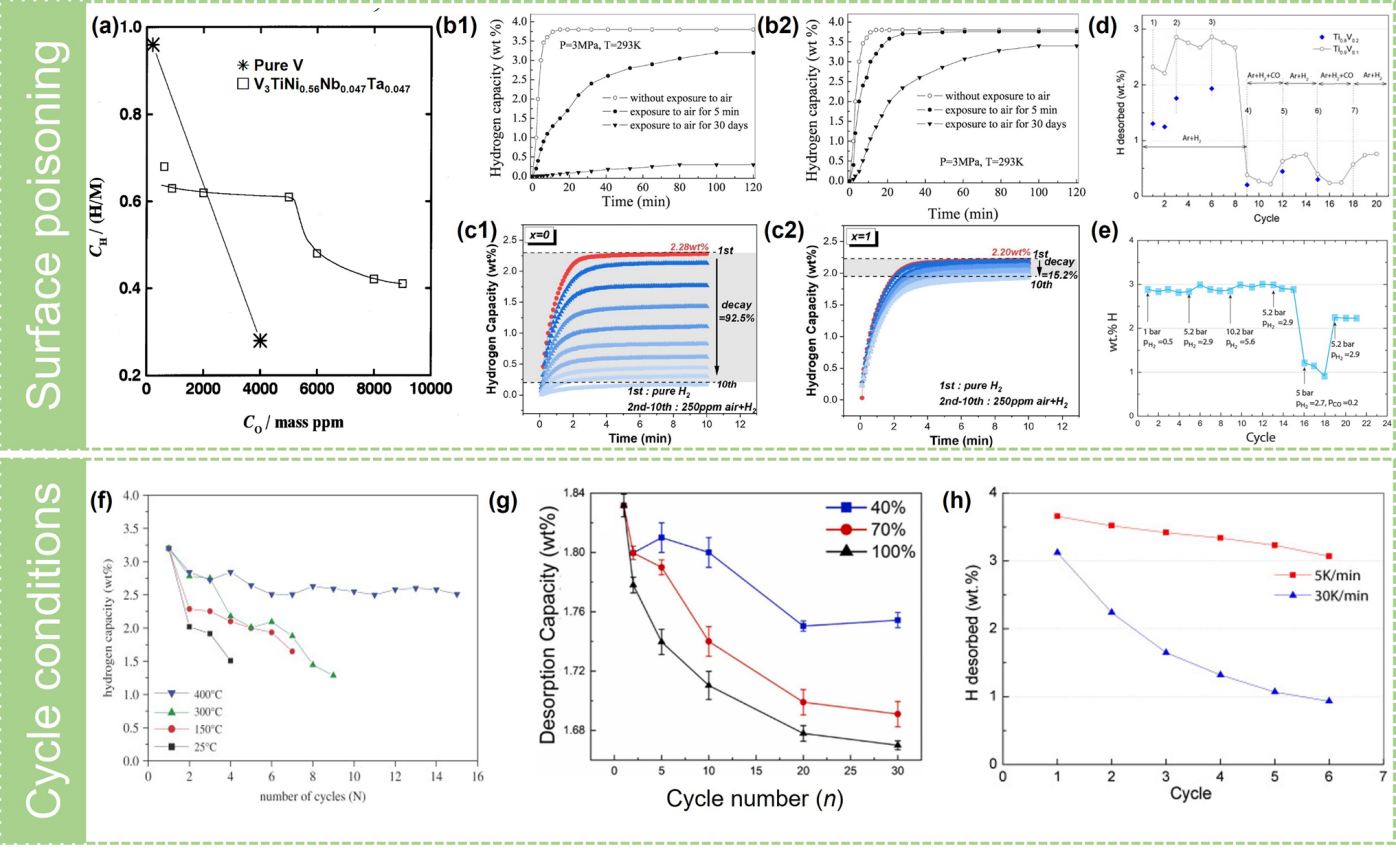
**a** Effective hydrogen capacity *C*_H_ plotted against the oxygen concentration Co for the V_3_TiNi_0.56_Co_0.14_Nb_0.047_Ta_0.047_ alloy in comparison with V. Reproduced with permission from Ref. [177]. Copyright 1998 Elsevier Hydrogen absorption curves of Ti–30V–15Mn–15Cr alloy powder with exposure to air for various durations: **b1** without nano ZnO and **b2** modified by 3 wt% nano ZnO. Reproduced with permission from Ref. [139]. Copyright 2004 Springer Nature. **c1** Hydrogen sorption cycling of the alloy *x* = 0 in H_2_ + 250 ppm air atmosphere and **c2** the alloy *x* = 1 in H_2_ + 250 ppm air atmosphere. Reproduced with permission from Ref. [173]. Copyright 2024 Elsevier **d** Amount of hydrogen desorbed during the TPR cycles. During hydrogen absorption, total gaseous pressure was 1 bar for both mixture I (Ar + H_2_) and II (Ar + H_2_ + CO) with a flow rate of 50 mL min^−1^. Hydrogen was absorbed during cooling to 200 °C and desorbed during heating of the samples to 700 °C with a rate of 30 K min^−1^ for both temperature increase and decrease. Composition of the mixtures during the absorption was as follows (1) Ar + 8%H_2_, (2) Ar + 50%H_2_, (3) Ar + 80%H_2_, (4) Ar + 50%H_2_ + 5%CO, (5) Ar + 5%H_2_, (6) Ar + 80%H_2_ + 2%CO, and (7) Ar + 8%H_2_. Desorption conditions were chosen as: 1 bar Ar gas; flow rate 50 mL min^−1^. Reproduced with permission from Ref. [33]. Copyright 2012 Elsevier **e** Reversible hydrogen storage capacity of Ti_0.9_V_0.1_ during hydrogenation and dehydrogenation cycling in the temperature range 150–780 °C, 5 deg min^−1^ cooling and heating rate. Hydrogenation was done at different hydrogen pressures, and dehydrogenation was done in pure Ar, 100 mL min^−1^ gas flow. CO was introduced into the gas flow before cycle 16 and removed after cycle 18. Reproduced with permission from Ref. [174]. Copyright 2016 Elsevier **f** Cyclic hydrogen capacities for the Ti_0.8_Cr_1.2_V alloy at various temperatures. Reproduced with permission from Ref. [175]. Copyright 2007 Elsevier **g** Desorption capacity at different DOD. Reproduced with permission from Ref. [176]. Copyright 2023 Elsevier **h** Amount of hydrogen desorbed from the Ti_0.9_V_0.1_–based hydride formed during H absorption at 1 bar Ar + 8%H_2_ at two different cooling rates, 5 and 30 K min^−1^. Gas flow rate was 30 mL min^−1^. Hydrogen desorption proceeded into Ar gas. Limiting absorption and desorption temperatures were 200 and 700 °C. Reproduced with permission from Ref. [33]. Copyright 2012 Elsevier

Some researchers have studied the capacity decay behavior of V–Ti-based hydrogen storage alloys in impure hydrogen. Generally, the influence of impurity oxygen atmosphere on pure V is greater than that of V–Ti alloys (Fig. 8a). The initial capacity of the V_40_Fe_8_Ti_26_Cr_26_ alloy decayed after four cycles in H_2_ blended with 250 ppm of O_2_, and the tolerance toward O_2_ of the alloy could be improved effectively by loading a layer of metal La on the surface of the alloy through surface engineering, which can provide diffusion pathways due to the formation of diffusion-inhibiting oxides [172]. Unfortunately, no specific hydrogen storage capacity was given. Forming the second phase in the BCC matrix is an effective strategy to improve the toxicity resistance. Yu et al. [139] investigated the effect of ZnO addition on hydrogen storage performance of the Ti-30V-15Mn-15Cr alloy after air exposure, as shown in Fig. 8b. The alloy with the addition of 3 wt% ZnO decreased sensitivity to air exposure since ZnO acted as a hydrogen diffusion channel for hydrogen into the bulk alloy, making the alloy easy to absorb hydrogen. In another study by Yu et al. [82], the Fe-substituted Ti–10Cr–18Mn–27V–5Fe was more easily activated than iron–free Ti–10Cr–18Mn–32V alloy after air exposure, which can be attributed to the fact that the C14 phase generated after Fe substitution was more likely to be cracked and formed more channels for H diffusion. Recently, Xie et al. [173] repoted a Si-induced air-tolerant hydride to improve air-poisoning resistance of vanadium-based alloys. The (V_75_Ti_11_Cr_13_Fe_1_)_99_Si_1_ alloy can maintain about 85% of hydrogen absorption capacity after 10 cycles in H_2_ + 250 ppm atmosphere, which can be contributed to the formation of Ti_5_Si_3_H_0.9_ induced by 1 at% Si addition during hydrogen absorption cycles, as shown in Fig. 8c. The Ti_5_Si_3_H_0.9_ played a role of hydrogen diffusion channel for the bulk. On the contrary, the Si-free V_75_Ti_11_Cr_13_Fe_1_ alloy almost lost the ability to absorb hydrogen under the same conditions. However, the addition of Si would inevitably reduce the reversible hydrogen storage capacity, which required a balance between toxic resistance and reversible hydrogen storage capacity. In addition, Suwarno et al. [33, 174] explored the possibility that the alloy could be reactivated after poisoning. When CO-containing gas was introduced during the hydrogen ab/desorption cycle, the hydrogen storage capacity would plummet. However, part of the hydrogen storage capacity would be restored when the CO-free hydrogen was re-introduced (Fig. 8d, e). This research, which can be defined as "waste alloy regeneration," contributed to the practical application of vanadium-based solid solution alloys.

The cycle conditions also affect the cyclic stability of the alloys to a certain extent. Lin et al. [175] reported the capacity durability of the Ti_0.8_Cr_1.2_V alloy at different temperatures for dehydrogenation and found that the cycle durability worsened with the decrease in the dehydrogenation temperature (Fig. 8f). The *β*-phase formed during hydrogen absorption had high thermal stability, but its reversibility was poor, requiring higher dehydrogenation temperatures, thus leading to a reduction of its room-temperature cycling stability [6]. Recently, Wu et al. [176] proposed that the depth of dehydrogenation (DOD) also affected cycle stability, and the capacity decay rate was 4.37% at 40% DOD but rose to 8.74% at 100% DOD (Fig. 8g). In practical applications, too much reduction in the depth of dehydrogenation may effectively reduce micro-strain accumulation and volume expansion. The heating rate during desorption process may also affect the cyclic properties of the alloys. In the study of Suwarno et al. [33], the heating rate of 30 K min^–1^ led to the obvious attenuation of hydrogen storage capacity compared with the heating rate of 5 K min^−1^ (Fig. 8h). Therefore, where possible, the establishment of a uniform cycle test standard will help to compare the cycle stability of different alloys.

### Some Issues of Using Industrial Grade V-Rich Alloy as a Raw Material for Large-Scale Applications

**Fig. 9:**
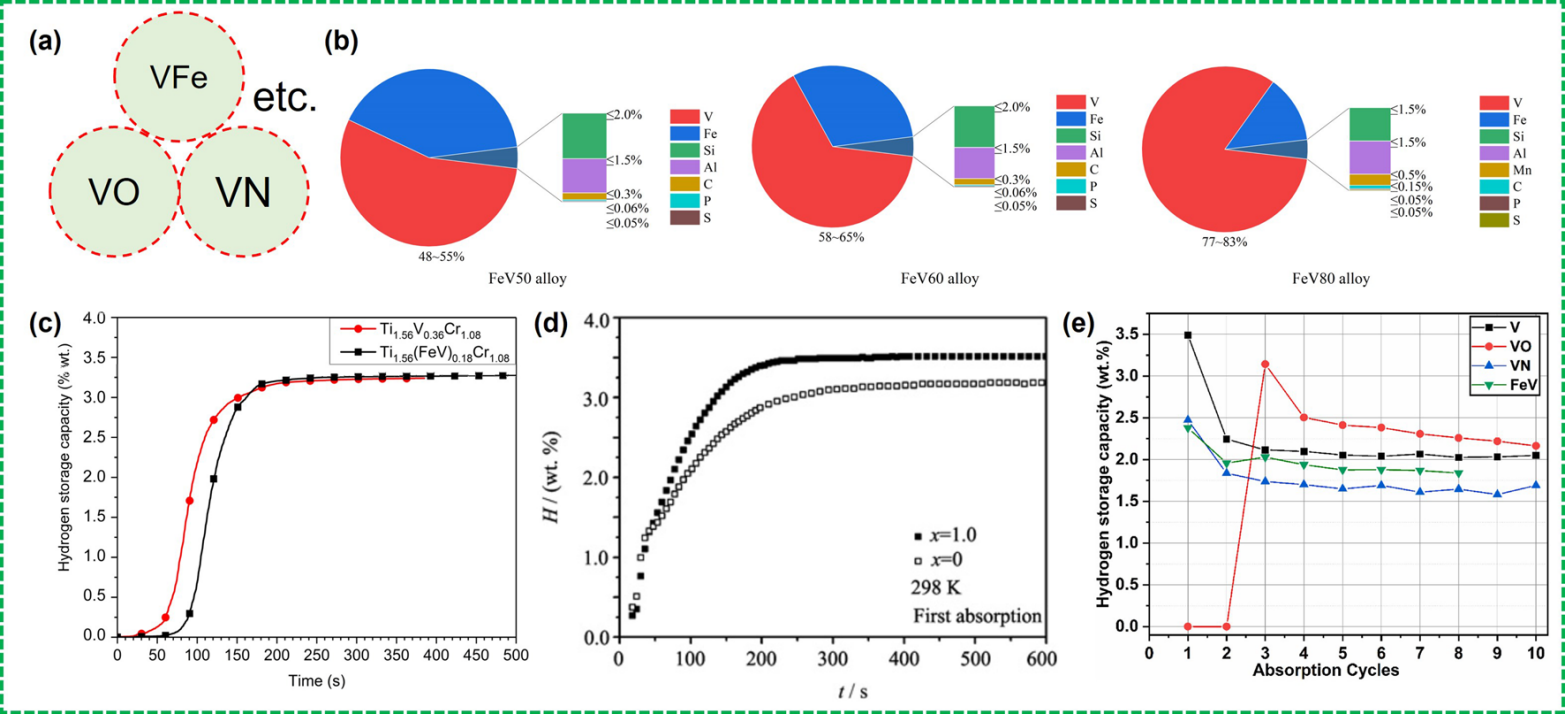
**a** Diagram of different types of vanadium alloys. **b** Pie chart of chemical composition of different ferrovanadium alloys (FeV50 alloy, FeV60 alloy, and FeV80 alloy in international standard ISO 5451:2022). **c** Activation kinetics of the co-melt Ti_1.56_V_0.18_(FeV)_0.18_Cr_1.08_ + 4%Zr_7_Ni_10_ and Ti_1.56_V_0.36_Cr_1.08_ + 4%Zr_7_Ni_10_ under 20 bars of H_2_ at 25 °C. Reproduced with permission from Ref. [182]. **d** First hydrogen absorption kinetics curves measured at 298 K for Ti_27.25_Cr_28.05_V_37.25_Fe_7.45_Ce_*x*_ (*x* = 0 and 1.0 at%) alloys. Reproduced with permission from Ref. [75]. Copyright 2010 Elsevier **e** Changes in the hydrogen storage capacity of all alloys with increasing cycles. Reproduced with permission from Ref. [184]. Copyright 2012 Elsevier

Due to the high costs of raw materials, especially for vanadium, replacing V with vanadium master alloy and using low-purity V are the main strategies to reduce the cost of preparing V-based alloys [50, 72, 73, 111, 130, 178]. There are many different types of industrial–grade vanadium alloys such as ferrovanadium (FeV) alloys, vanadium nitride (VN) alloys, and vanadium oxygen (VO) alloys (Fig. 9a). Among them, the residual B, C, N, O, Si, and Al, etc., in the raw metal vanadium or V alloys will have a key impact on the performance of the prepared V–Ti-based solid solution alloys [103, 178–181]. Ferrovanadium alloys have been the most studied in cost reduction possibilities for the preparation of V–Ti-based solid solution alloys. Fig. 9b shows the chemical composition requirements of different ferrovanadium alloys in international standard (ISO 5451:2022). The Chinese standards for ferrovanadium alloys provide more detailed criteria for composition control and different grades. Understanding the effect of impurity elements in ferrovanadium alloys on hydrogen storage properties is the key to prepare high-performance and low-cost V–Ti-based solid solution alloys.

One of the consequences of using ferrovanadium alloy instead of partial pure vanadium as raw material is that the incubation time of the first hydrogenation would be increased. Hout et al. [182] replaced 50% vanadium by ferrovanadium to prepare a low-cost V–Ti-based BCC alloy. As can be seen from Fig. 9c, the FeV substituted Ti_1.56_V_0.18_(FeV)_0.18_Cr_1.08_ + 4%Zr_7_Ni_10_ alloy experienced a longer incubation time during the first hydrogenation process than the unsubstituted Ti_1.56_V_0.36_Cr_1.08_ + 4%Zr_7_Ni_10_ alloy at 25 °C. The oxygen content in raw materials has a key effect on the hydrogen storage properties of V–Ti-based hydrogen storage alloys. For example, the elements with a higher affinity toward oxygen, e.g., Ti, would promote the formation of a Ti–rich secondary phase that led to an increase in the pressure of the plateau [107, 180]. Deoxidization can be achieved by the addition of La, Y, and Ce and further improve the flatness of plateau and enlarge the hydrogen capacity [75]. Mi et al. [75] prepared a low-cost V–Ti-based BCC alloy with commercial ferrovanadium and the Ti_27.25_Cr_28.05_V_37.25_Fe_7.45_Ce_1_ alloy exhibited a faster first hydrogenation kinetics with 1 at% Ce added, as shown in Fig. 9d. In addition, Ce substitution was shown to improve the activation performance of the Ti_33_V_37_Mn_30_ alloy due to the formation of CeH_2.51_, and the hydrogen absorption capacity of the alloy increased with the increase of Ce content [40]. But unfortunately, the introduction of Ce led to the increase of the stability of the formed hydride, which resulted in a relatively high temperature needed to release H_2_.

The effect of the Al element on the hydrogen storage properties of V–Ti-based alloys has been explored in many studies [39, 93, 104–106]. The V_48_Fe_12_Ti_15_Cr_25_ alloy exhibited a lower desorption kinetics performance and higher thermodynamic stability with 1 at% Al substitution [93]. Mi et al. [104] studied the effect of Al addition on the microstructures and hydrogen storage properties of Ti_26.5_Cr_20_(V_0.45_Fe_0.085_)_100−*x*_Al_*x*_Ce_0.5_ (*x* = 0, 0.5, 1.0, and 1.5 at%) alloys. They found that with the increase of Al content, the lattice parameters of the BCC phase, desorption plateau pressure and slope factor of the alloys increased, but the hydrogen storage capacity decreased. Ce is also a useful element to suppress the side effect of Al on the hydrogen storage performance of the Ti–V alloy, which could format the Al–Ce–O secondary phase. However, Wu et al. [39] reported that the simultaneous introduction of Al and Fe into the V–Ti–Cr alloy resulted in better hydrogen storage properties than only adding Fe or Al. They believe that Al preferentially occupies the position along *c* axis in the V-based solid solution alloy that leads to a decrease in the lattice parameter of *c*, while the addition of Fe will shorten the lattice parameter a but extend the c. The overall result is that the change in lattice volume would be canceled out.

Similarly, the Si element also adversely affects the hydrogen storage properties of V–Ti-based solid solution alloys [77, 103, 183]. Although Si addition could improve the activation property due to the appearance of the secondary Laves phase, the hydrogen absorption and desorption capacities were found to have been decreased while the plateau slope factor and pressure were increased [77]. Earlier, we also mentioned that the right amount of silicon could improve the ability to resist poisoning [173]. It is undeniable that the loss of reversible hydrogen storage capacity is inevitable. Huang et al. [103] reported similar results that Si element gave rise to the precipitation of a C14 TiFe_2_-type Laves phase, resulting in the inhomogeneity of the chemical composition for the Ti_26.5_V_0.45_Fe_8.5_Cr_20_Ce_0.5_ alloy. Ce could eliminate the negative effects brought by Si, but the mechanism involved is still unclear [183]. The increase of B content will also lead to a decrease of the hydrogen storage capacity of the alloy [61]. Therefore, it is necessary to remove impurities from raw materials.

Several years ago, Chen et al. [73] successfully prepared a V_30_Ti_32_Cr_32_Fe_6_ alloy from FeV80 alloy that could release 2.35 and 2.56 wt% hydrogen at 298 and 373 K, respectively. Recently, Sharma and Bishnoi compared the hydrogen storage properties of V-base solid solution alloys prepared with different vanadium raw materials (pure V, FeV, VN, and VO). As shown in Fig. 9e, the first hydrogenation process of the 52Ti–12VO–36Cr + 4 wt%Zr alloy prepared by VO alloy did not absorb hydrogen, which was significantly different from the alloy prepared using pure V with no apparent incubation period [184]. This was because that the VO alloy contained 3.4% oxygen, which led to the formation of an oxide layer on the surface of the alloy after melting that impeded the hydrogenation process. However, after two activations, the 52Ti–12VO–36Cr + 4 wt%Zr alloy prepared by VO showed a high hydrogen storage capacity, which was comparable to that of alloy prepared by pureV. This implies the possibility of using low-cost vanadium raw materials to prepare high-performance V–Ti-based solid solution alloys. As we mentioned before, most of the impurity elements in raw materials can be effectively removed by adding metal Ce. On this base, under the premise of using FeV master alloy for achieving large-scale application, it is imperative to improve the output of a single furnace and reduce the composition difference of different batches while ensure the quality stability of V–Ti-based solid solution alloys.

## Conclusion and Outlook

V–Ti-based solid solution alloys are promising candidates for solid-state metal hydride tanks, which operate under non-extreme temperatures and pressures and supply hydrogen to fuel cells. These alloys, primarily consisting of a body-centered cubic main phase, have attracted much attention due to their high hydrogen storage density at room temperature and adjustable plateau pressure. To explore the application of V–Ti-based solid solution alloys in the field of hydrogen storage, extensive efforts have been initiated, including the optimization of preparing technologies, compositional adjustments, heat treatments, quenching techniques, impurity analysis, and understanding of degradation mechanism. So far, arc melting is still the preferred method for preparing V–Ti solid solution alloys, and V–Ti–Cr and V–Ti–Fe alloys are the two most studied systems. On this basis, the hydrogen storage properties of V–Ti-based solid solution alloys can be improved by adjusting the proportions of V, Ti, Nb, Fe, Ni, Zr, Ce, and other elements. Heat treatment can flatten the plateau and increase reversible hydrogen storage capacity. Although various alloys with excellent hydrogen storage properties have been developed, a reversible hydrogen storage capacity of 2.5 wt% and above seems to be relatively difficult at near-room temperature. And the efficient and stable preparation of V–Ti-based solid solution alloys on large-scale is also a challenge besides the problem of high cost.

To date, many studies only focus on the influence of elements on one aspect of hydrogen storage performance (e.g., activation property) for V–Ti-based solid solution alloys. For practical applications, a modified strategy should be able to address the performance of one aspect while maintaining the performance of other aspects. There are many balances to be considered, such as "ease in activation" vs "high reversible hydrogen storage capacity," "anti-poisoning" and "cost" vs "hydrogen storage capacity."

High vanadium content alloys are currently considered the best option for achieving high reversible hydrogen storage capacity and excellent cyclic stability. The viable method involves the using of a FeV master alloy instead of metallic vanadium, although the impurity removal process still requires further enhancement. Therefore, developing V–Ti-based solid solution alloys that are cost-effective with high reversible capacities requires relentless effort, particularly for the application of solid-state MH tanks.
